# Pediatric vs Adult or Mixed Trauma Centers in Children Admitted to Hospitals Following Trauma: A Systematic Review and Meta-Analysis

**DOI:** 10.1001/jamanetworkopen.2023.34266

**Published:** 2023-09-18

**Authors:** Lynne Moore, Gabrielle Freire, Alexis F. Turgeon, Mélanie Bérubé, Khadidja Malloum Boukar, Pier-Alexandre Tardif, Henry T. Stelfox, Suzanne Beno, François Lauzier, Marianne Beaudin, Roger Zemek, Isabelle J. Gagnon, Emilie Beaulieu, Matthew John Weiss, Sasha Carsen, Belinda Gabbe, Antonia Stang, Anis Ben Abdeljelil, Eunice Gnanvi, Natalie Yanchar

**Affiliations:** 1 Population Health and Optimal Health Practices Research Unit, Trauma–Emergency–Critical Care Medicine, Centre de Recherche du CHU de Québec–Université Laval (Hôpital de l’Enfant-Jésus), Québec City, Québec, Canada; 2 Department of Social and Preventative Medicine, Université Laval, Québec, Québec, Canada; 3 Division of Emergency Medicine, Department of Pediatrics, Faculty of Medicine, University of Toronto, Toronto, Ontario, Canada; 4 Department of Anesthesiology and Critical Care Medicine, Division of Critical Care Medicine, Université Laval, Québec City, Québec, Canada; 5 Faculty of Nursing, Université Laval, Québec City, Québec, Canada; 6 Departments of Critical Care Medicine, Medicine and Community Health Sciences, O’Brien Institute for Public Health, University of Calgary, Calgary, Alberta, Canada; 7 Division of Emergency Medicine, Hospital for Sick Children, University of Toronto, Toronto, Ontario, Canada; 8 Sainte-Justine Hospital, Department of Paediatric Surgery, Université de Montréal, Montréal, Québec, Canada; 9 Department of Pediatrics, Children’s Hospital of Eastern Ontario, Ottawa, Ontario, Canada; 10 Division of Pediatric Emergency Medicine, McGill University Health Centre, Montreal Children’s Hospital, Montréal, Québec, Canada; 11 School of Physical and Occupational Therapy, Faculty of Medicine and Health Sciences, McGill University, Montréal, Québec, Canada; 12 Département de pédiatrie, Faculté de médecine, Centre Hospitalier Universitaire de Québec-Université Laval, Québec City, Québec, Canada; 13 Centre Mère-Enfant Soleil du CHU de Québec, Transplant Québec, Québec, Québec, Canada; 14 Division of Orthopaedic Surgery, Children’s Hospital of Eastern Ontario, Ottawa, Ontario, Canada; 15 School of Public Health and Preventive Medicine, Monash University, Melbourne, Victoria, Australia; 16 Pediatrics, Emergency Medicine, and Community Health Sciences, Cumming School of Medicine, University of Calgary, Calgary, Alberta, Canada; 17 Department of Surgery, University of Calgary, Calgary, Canada

## Abstract

**Importance:**

Adult trauma centers (ATCs) have been shown to decrease injury mortality and morbidity in major trauma, but a synthesis of evidence for pediatric trauma centers (PTCs) is lacking.

**Objective:**

To assess the effectiveness of PTCs compared with ATCs, combined trauma centers (CTCs), or nondesignated hospitals in reducing mortality and morbidity among children admitted to hospitals following trauma.

**Data Sources:**

MEDLINE, Embase, and Web of Science through March 2023.

**Study Selection:**

Studies comparing PTCs with ATCs, CTCs, or nondesignated hospitals for pediatric trauma populations (aged ≤19 years).

**Data Extraction and Synthesis:**

This systematic review and meta-analysis was performed following the Preferred Reporting Items for Systematic Review and Meta-analysis and Meta-analysis of Observational Studies in Epidemiology guidelines. Pairs of reviewers independently extracted data and evaluated risk of bias using the Risk of Bias in Nonrandomized Studies of Interventions tool. A meta-analysis was conducted if more than 2 studies evaluated the same intervention-comparator-outcome and controlled minimally for age and injury severity. Subgroup analyses were planned for age, injury type and severity, trauma center designation level and verification body, country, and year of conduct. Grading of Recommendations Assessment, Development, and Evaluation (GRADE) was used to assess certainty of evidence.

**Main Outcome(s) and Measure(s):**

Primary outcomes were mortality, complications, functional status, discharge destination, and quality of life. Secondary outcomes were resource use and processes of care, including computed tomography (CT) and operative management of blunt solid organ injury (SOI).

**Results:**

A total of 56 studies with 286 051 participants were included overall, and 34 were included in the meta-analysis. When compared with ATCs, PTCs were associated with a 41% lower risk of mortality (OR, 0.59; 95% CI, 0.46-0.76), a 52% lower risk of CT use (OR, 0.48; 95% CI, 0.26-0.89) and a 64% lower risk of operative management for blunt SOI (OR, 0.36; 95% CI, 0.23-0.57). The OR for complications was 0.80 (95% CI, 0.41-1.56). There was no association for mortality for older children (OR, 0.71; 95% CI, 0.47-1.06), and the association was closer to the null when PTCs were compared with CTCs (OR, 0.73; 95% CI, 0.53-0.99). Results remained similar for other subgroup analyses. GRADE certainty of evidence was very low for all outcomes.

**Conclusions and Relevance:**

In this systematic review and meta-analysis, results suggested that PTCs were associated with lower odds of mortality, CT use, and operative management for SOI than ATCs for children admitted to hospitals following trauma, but certainty of evidence was very low. Future studies should strive to address selection and confounding biases.

## Key Points

**Question:**

Do children admitted to hospitals following trauma who receive definitive treatment in a pediatric trauma center (PTC) have better outcomes than those treated in adult trauma centers (ATCs)?

**Findings:**

Results of this systematic review and meta-analysis of 56 studies with 286 051 participants suggest that compared with ATCs, PTCs were associated with a reduction of 41%, 52%, and 64% in the odds of mortality, computed tomography, and operative management for blunt solid organ injury, respectively, for children hospitalized following trauma. Certainty of evidence was very low.

**Meaning:**

These results suggest treating children in PTCs leads to better outcomes, but future studies should strive to address selection and confusion biases.

## Teaser

This systematic review and meta-analysis evaluates whether children admitted to hospitals following trauma who receive definitive treatment in a pediatric trauma center have better outcomes than those treated in adult trauma centers.

## Introduction

Injury is the medical condition with the greatest burden on US children and youth.^1^ In the US, 4.1 million children get injured every year at an annual cost of US $396 billion.^2^ The human and societal burden of childhood injury are even greater. For every injury fatality, 10 children are left with lifelong disabilities resulting in enormous emotional and financial hardship for the injured and their families.^3^

Trauma systems constitute a population-based, multidisciplinary response to injury from prehospital and hospital care to rehabilitation and community services and have been shown to improve patient outcomes.^4,5,6,7,8^ Systematic reviews provide evidence that adults treated at designated trauma centers have better outcomes than those treated in nondesignated hospitals.^5,9,10^ The first pediatric trauma centers (PTCs) were established in the 1970s,^11^ soon after the creation of adult trauma centers (ATCs), in recognition of the distinct physiological, psychological, and social needs of children.^12^ Studies comparing outcomes of injured children treated at PTCs, ATCs, or combined (adult and pediatric) trauma centers (CTCs) have shown divergent results,^13,14,15,16,17,18,19^ and a synthesis of this evidence is lacking.

Our primary objective was to synthesize evidence on the effectiveness of PTCs compared with ATCs, CTCs, or nondesignated hospitals for improving clinical outcomes and quality of life in children admitted to hospitals following trauma. Secondary objectives were to assess associations with resource utilization and processes of care. We hypothesized that children receiving definitive treatment in a PTC would have more favorable outcomes than those treated in other hospitals.

## Methods

We conducted this systematic review following Cochrane methodology^20^ and reported results according to the Meta-analysis of Observational Studies in Epidemiology (MOOSE) reporting guideline^21^ and Preferred Reporting Items for Systematic Reviews and Meta-analyses (PRISMA) reporting guideline.^22^ The protocol was registered with PROSPERO. The study was designed and conducted in collaboration with an interdisciplinary advisory committee comprising 12 pediatric clinicians (including prehospital, emergency medicine, trauma surgery, neurosurgery, orthopedics, critical care medicine, nursing, and rehabilitation specialties), 3 trauma program medical directors, and 2 trauma accreditation agency representatives.

### Inclusion Criteria and Search Strategy

We included prospective or retrospective cohort and case-control or case-cohort studies that compared outcomes of children or adolescents (aged ≤19 years) admitted to hospitals following trauma in PTCs to those in ATCs, CTCs, or nondesignated hospitals (see eTable 1 in Supplement 1 for definitions). We searched MEDLINE, Embase, Web of Science, and CINAHL through March 2023 (eTable 2 in Supplement 1). Thesis repositories and references of included studies were screened. Our primary outcomes of interest, defined a priori, were mortality, complications, functional status, discharge destination, and quality of life (see eTable 3 in Supplement 1 for definitions). Secondary outcomes were resource utilization and processes of care including use of computed tomography (CT) or operative management of blunt solid organ injuries (SOI).

### Risk of Bias Assessment

Three content experts (L.M., P.A.T., J.G.) independently rated studies using the Risk of Bias in Nonrandomized Studies of Interventions tool.^23^ We assessed publication bias using a contour-enhanced funnel plot and estimated the magnitude of the potential bias with the trim-and-fill method.^24^

### Statistical Analysis

#### Data Synthesis

We restricted data synthesis to studies presenting measures of association minimally adjusted for age and injury severity. In the context of a review limited to observational studies with a very high risk of indication bias, unadjusted comparisons were considered not to produce meaningful results. When 2 studies or more evaluated the same exposure-outcome association, we conducted meta-analyses (see eAppendix 1 in Supplement 1). We measured the heterogeneity of included studies using the *I^2^* statistic and interpreted as low if 0% to 40%, moderate if 30% to 60%, substantial if 50% to 90%, and considerable if 75% to 100%.^20^ Two content experts (L.M., P.A.T.) independently applied Grading of Recommendations Assessment, Development and Evaluation criteria (eAppendix 1 in Supplement 1).^25^

#### Subgroup and Sensitivity Analyses

We conducted prespecified subgroup analyses for factors thought to modify the associations of interest, identified on consultation with our advisory committee: age, type of injury, injury severity, country, year of study conduct, PTC and ATC designation levels and verification body, and risk of bias (see eTable 4 in Supplement 1 for definitions). We added post-hoc subgroup analyses on how transfers were handled in analyses, as this was felt to be a major source of potential bias. We conducted sensitivity analyses for outliers.^26^ All analyses were conducted using R Statistical Software version 4.2.1 (R Project for Statistical Computing) and statistical significance was set at P < 0.05.

## Results

Among 6860 records identified in the databases, 5369 titles and abstracts were screened, and 212 manuscripts were assessed for eligibility, of which 56,^4,8,12,13,14,18,19,27,28,29,30,31,32,33,34,35,36,37,38,39,40,41,42,43,44,45,46,47,48,49,50,51,52,53,54,55,56,57,58,59,60,61,62,63,64,65,66,67,68,69,70,71,72,73,74,75^ with 286 051 total participants, were included in the review (eFigure 1 in eAppendix 2 in Supplement 1). Forty-four studies (79%) were published after 2010 (eTable 5 in Supplement 1). Forty-three studies (77%) were conducted in the USA, 3 in Australia, and 2 in Canada with recruitment periods spanning from 1985 to 2019. Forty-seven studies (84%) compared PTCs with ATCs, and 18 compared PTCs with CTCs. Only 1 compared PTCs with nondesignated hospitals.^61^ Thirty-nine studies (76%) presented measures of association minimally adjusted for age and injury severity.

### Risk of Bias

Overall risk of bias was critical in 6 studies^13,32,35,18,54,70^ (25%) for mortality, critical in all studies for complications, serious in all studies for CT use, and serious in 5 studies^31,52,54,64,69^ (71%) for operative management of SOI (Table 1). Selection bias was rated critical or serious in 28^8,13,14,18,27,29,31,32,33,34,35,38,41,50,54,55,57,58,62,63,67,69,70^ studies (82%), either because participation in the trauma registry was voluntary or because a substantial proportion of patients were excluded (eg, missing data or patients transferred in). Confounding bias was rated serious for 11 studies^13,29,47,52,53,58,59,60,63,64,67^ (17%), mostly due to the absence of physiological parameters (eg, Glasgow coma scale or hemodynamic stability) in administrative data. Bias due to classification of the intervention was rated low for 27 studies^4,13,14,18,27,29,31,33,38,41,50,67,52,53,54,57,59,60,63,64,65,66,69,70^ (79%), with some studies rated moderate because trauma centers were not accredited or verified by the same organizations (eg, state-designated with or without American College of Surgeons [ACS] verification). Similarly, deviations from the intervention were rated low for 26 studies^4,14,18,29,31,32,33,35,38,41,47,50,54,55,57,58,59,62,65,66^ (76%), with 11 rated moderate because they included but did not control for interhospital transfers. Bias in the measurement of outcomes was rated low for mortality, CT, and surgery but critical for complications, vulnerable to misclassification.^76^ Bias in reported results was rated serious for 13 studies^4,8,14,27,32,33,35,47,50,55,59,62^ (38%), which conducted unplanned subgroup analysis. Finally, a small-study effect (proxy for publication bias) was observed for mortality with an adjusted 95% CI covering the null value (OR, 0.79; 95% CI, 0.58-1.06) (eFigure 2 in Supplement 1).

**Table 1.** Risk Of Bias of Studies Included in Meta-Analyses^a^

Outcome and source	Confounding	Selection	Classification of intervention	Deviations from interventions	Missing data	Measurement of outcomes	Reported results	Overall
Mortality								
Derderian et al,^13^ 2022	Serious	Critical	Low	Moderate	NI	Low	Moderate	Critical
Lewit et al,^29^ 2022	Serious	Serious	Low	Low	Serious	Low	Moderate	Serious
Pulido et al,^31^ 2022	Moderate	Serious	Low	Low	NI	Low	Moderate	Serious
Sheff et al,^32^ 2022	Moderate	Critical	Moderate	Low	Serious	Low	Serious	Critical
Stephenson et al,^33^ 2022	Moderate	Serious	Low	Low	NI	Low	Serious	Serious
Ali et al,^34^ 2021	Moderate	Serious	Moderate	Moderate	Moderate	Low	Moderate	Serious
Evans et al,^14^ 2021	Moderate	Serious	Low	Low	Moderate	Low	Serious	Serious
Khalil et al,^35^ 2021	Moderate	Critical	Moderate	Low	Serious	Low	Serious	Critical
Scantling et al,^38^ 2021	Moderate	Serious	Low	Low	Serious	Low	Moderate	Serious
Swendiman et al,^18^ 2021	Moderate	Critical	Low	Low	Moderate	Low	Moderate	Critical
Hatchimonji et al,^41^ 2020	Moderate	Serious	Low	Low	Moderate	Low	Moderate	Serious
Myers et al,^47^ 2019	Serious	Low	Moderate	Low	NI	Low	Serious	Serious
Bardes et al,^50^ 2018	Moderate	Serious	Low	Low	NI	Low	Serious	Serious
Mitchell et al,^67^ 2017	Serious	Serious	Low	Moderate	Low	Low	Moderate	Serious
Miyata et al,^54^ 2017	Moderate	Serious	Low	Low	NI	Low	Moderate	Serious
Miyata et al,^55^ 2017	Moderate	Critical	Moderate	Low	NI	Low	Serious	Critical
Walther et al,^63^ 2016	Serious	Serious	Low	Moderate	NI	Low	Moderate	Serious
Webman et al,^8^ 2016	Moderate	Serious	Moderate	Low	Moderate	Low	Serious	Serious
Sathya et al,^62^ 2015	Moderate	Serious	Moderate	Low	Moderate	Low	Serious	Serious
Walther et al,^59^ 2014	Serious	Low	Low	Low	NI	Low	Serious	Serious
Matsushima et al,^65^ 2013	Moderate	Moderate	Low	Low	NI	Low	Moderate	Moderate
Mitchell et al,^53^ 2013	Serious	Low	Low	Moderate	NI	Low	Moderate	Serious
Amini et al,^4^ 2011	Moderate	Low	Low	Low	Moderate	Low	Serious	Serious
Osler et al,^70^ 2001	Moderate	Critical	Low	Moderate	NI	Low	Moderate	Critical
Complications								
Ali et al,^34^ 2022	Moderate	Serious	Moderate	Moderate	Moderate	Critical	Moderate	Critical
Khalil et al,^35^ 2021	Moderate	Critical	Moderate	Low	Serious	Critical	Serious	Critical
Matsushima et al,^65^ 2013	Moderate	Moderate	Low	Low	NI	Critical	Moderate	Critical
Computed tomography								
Gerber et al,^27^ 2023	Moderate	Serious	Low	Low	Serious	Low	Serious	Serious
Ali et al,^34^ 2022	Moderate	Serious	Moderate	Moderate	Moderate	Low	Moderate	Serious
Pandit et al,^57^ 2016	Moderate	Serious	Low	Low	NI	Low	Moderate	Serious
Kelley-Quon et al,^60^ 2015	Serious	Low	Low	Serious	NI	Low	Moderate	Serious
Operative management of solid organ injury								
Pulido et al,^31^ 2022	Moderate	Serious	Low	Low	NI	Low	Moderate	Serious
Adams et al,^52^ 2017	Serious	Low	Low	Moderate	NI	Low	Moderate	Serious
Miyata et al,^54^ 2017	Moderate	Serious	Low	Low	NI	Low	Moderate	Serious
Safavi et al,^58^ 2016	Serious	Critical	Moderate	Low	Serious	Low	Moderate	Critical
Lippert et al,^64^ 2013	Serious	Moderate	Low	Moderate	NI	Low	Moderate	Serious
Matsushima et al,^66^ 2013	Moderate	Moderate	Low	Low	NI	Low	Moderate	Moderate
Potoka et al,^69^ 2002	Moderate	Serious	Low	Moderate	NI	Low	Moderate	Serious

Abbreviation: NI, no information.

^a^ Using the Risk Of Bias In Nonrandomized Studies of Interventions tool.

### PTCs vs ATCs

#### Mortality

PTCs were associated with 40% lower odds of mortality than ATCs (23 studies; OR, 0.59; 95% CI, 0.46-0.76) (Table 2 and Figure). However, statistical heterogeneity was considerable (*I^2^* = 78%) and prediction intervals included nonbeneficial effect estimates.

**Table 2.** Summary of Meta-Analyses Results for Children Admitted to Hospitals Following Trauma Treated at a Pediatric Trauma Center vs an Adult Trauma Center or a Combined Adult and Pediatric Center

Outcome	Pooled effect estimates OR (95% CI; 95% PI)	No. (*I^2^, %*)	Risk of bias^a^
Mortality			
PTC vs ATC, all	0.59 (0.46-0.76; 0.22-1.59)	23 (78)	Serious
Aged ≤16 y	0.50 (0.31-0.81; 0.15-1.71)	9 (64)	Serious
Aged ≥14 y	0.71 (0.47-1.06; 0.21-2.38)	11 (76)	Serious
Head injuries	0.66 (0.51-0.84; 0.08-5.13)	3 (<1)	Serious
Blunt solid organ injuries	0.67 (0.03-12.79; NA)	3 (91)	Serious
Penetrating injuries	0.48 (0.35-0.67; 0.30-0.77)	4 (<1)	Serious
ISS ≥12	0.60 (0.16-2.25; NA)	3 (77)	Serious
Level I	0.48 (0.30-0.76; 0.13-1.72)	8 (87)	Serious
Level I and II	0.56 (0.41-0.76; 0.34-0.93)	10 (44)	Serious
Level I and II with same verification organization	0.38 (0.20-0.75; 0.09-1.57)	4 (34)	Serious
US	0.68 (0.53-0.86; 0.29-1.57)	19 (77)	Serious
Other countries	0.27 (0.15-0.48; 0.11-0.69)	4 (<1)	Serious
2010 to 2014^b^	0.65 (0.45-0.94; 0.26-1.62)	10 (74)	Serious
2015 to 2019^b^	0.60 (0.37-0.99; 0.14-2.58)	10 (81)	Serious
Moderate or serious risk of bias^c^	0.53 (0.41-0.69; 0.23-1.25)	18 (76)	Serious
Critical risk of bias	0.80 (0.36-1.76; 0.10-6.42)	5 (84)	Serious
Transfers excluded	0.58 (0.47-0.71; 0.34-0.99)	14 (49)	Serious
Transfers included, adjustment	0.70 (0.40-1.22; 0.30-1.60)	4 (41)	Serious
Transfers included, no adjustment	0.42 (0.12-1.45; 0.01-12.62)	5 (91)	Serious
PTC vs CTC	0.73 (0.53-0.99; 0.36-1.46)	1 (69)	Serious
Complications			
PTC vs ATC, all	0.80 (0.41-1.56; 0.03-24.19)	3 (69)	Critical
Level I and II	0.80 (0.41-1.56; 0.03-24.19)	3 (69)	Serious
Computed tomography imaging			
PTC vs ATC, all	0.48 (0.26-0.89; 0.08-2.75)	7 (96)	Serious
Operative management of blunt solid organ injury			
PTC vs ATC, all	0.36 (0.23-0.57; 0.10-1.29)	8 (76)	Serious
Level I	0.24 (0.24-0.24; NA)	2 (NA)	Serious
Level I and II	0.38 (0.21-0.69; 0.09-1.71)	6 (79)	Serious
Level I and II with same verification organization	0.59 (0.03-12.30; NA)	2 (20)	Serious

Abbreviations: ATC, adult trauma centers; CTC, combined trauma centers; ISS, injury severity score; NA, not applicable; PI, prediction intervals; PTC, pediatric trauma centers.

^a^ Using the Risk of Bias in Nonrandomized Studies of Interventions tool.

^b^ Dates correspond to last year of patient recruitment.

^c^ One study with moderate and 17 with serious risk of bias.

**Figure.**
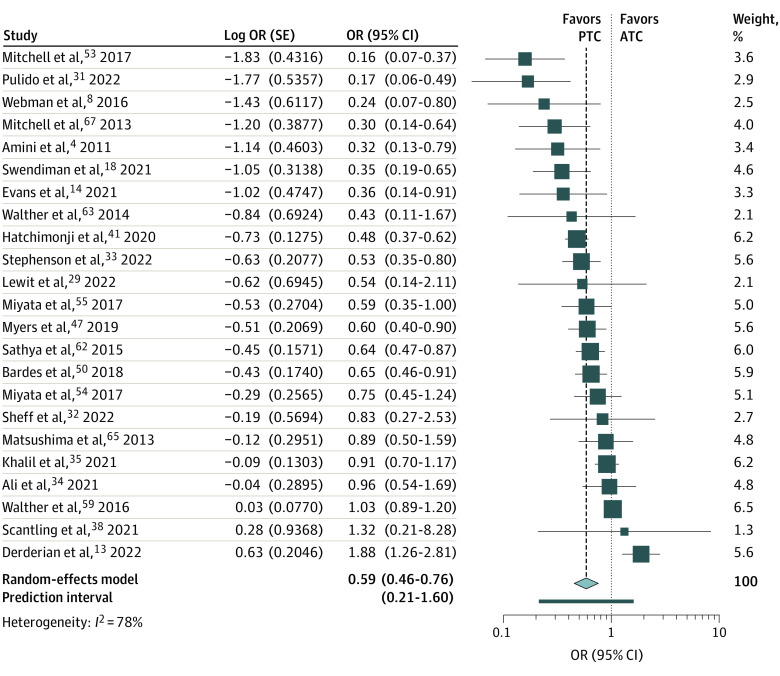
Forest Plots Describing the Odds of Mortality for Children Admitted to Hospitals Following Trauma Treated at Pediatric Trauma Centers (PTCs) vs Adult Trauma Centers (ATCs)

ORs of mortality for PTC vs ATC were comparable to the main analysis for children aged 16 years or younger (9 studies; OR, 0.50; 95% CI, 0.31-0.81) (Table 2; eFigure 3 in Supplement 1), but the 95% CI covered the null value for older adolescents (11 studies; OR, 0.71; 95% CI, 0.47-1.06) (Table 2; eFigure 4 in Supplement 1). Analyses by type of injury showed lower mortality at PTCs when compared with ATCs for head injury (3 studies; OR, 0.66; 95% CI, 0.51-0.84) (Table 2; eFigure 5, eFigure 6, eFigure 7, and eFigure 8 in Supplement 1) and penetrating injuries (4 studies; OR, 0.48; 95% CI, 0.35-0.67). Analyses for blunt SOI and injury severity scores (ISS) of 12 or higher led to low sample sizes with wide confidence intervals, but effect estimates were similar to the main analysis. When we restricted to studies with the same designation level in intervention and control groups and to trauma centers verified by the same organizations, results were similar (OR, 0.48; 95% CI, 0.30-0.76 for level I; OR, 0.56; 95% CI, 0.41-0.6 for levels I and II; and OR, 0.38; 95% CI, 0.20-0.75 for centers verified by the same organization) (Table 2; eFigure 9, eFigure 10, and eFigure 11 in Supplement 1). Subgroup analyses by country and by period of data collection also led to similar effect estimates (OR, 0.68; 95% CI, 0.53-0.86 for studies conducted in the US; OR, 0.27; 95% CI, 0.15-0.48 for other countries; OR, 0.65; 95% CI, 0.45-0.94 for 2010 to 2014; and OR, 0.60; 95% CI, 0.37-0.99 for 2015 to 2019) (Table 2; eFigure 12, eFigure 13, eFigure 14, and eFigure 15 in Supplement 1). Results remained unchanged in studies at moderate or serious risk of bias (OR, 0.53; 95% CI, 0.41-0.69) but the difference was not significant in studies at critical risk of bias (OR, 0.80; 95% CI, 0.36-1.76) (Table 2; eFigure 16 and eFigure 17 in Supplement 1). Subgroup analyses according to how transfers were handled led to similar ORs but were only significant in studies that excluded transfers (OR, 0.58; 95% CI, 0.47-0.71; OR, 0.70; 95% CI, 0.40-1.22 for studies including transfers with adjustment; and OR, 0.42; 95% CI, 0.12-1.45 for studies including transfers without adjustment) (Table 2; eFigure 18, eFigure 19, and eFigure 20 in Supplement 1). Removing outliers from the main analysis did not alter the conclusions (OR, 0.68; 95% CI, 0.57-0.81 when 7 studies were removed; and OR, 0.57; 95% CI, 0.46-0.71 when 3 studies were removed) (eFigure 21 and eFigure 22 in Supplement 1) and led to a reduction in heterogeneity (*I^2^* = 21% and *I^2^* = 54%, respectively).

### Complications

The confidence interval of the pooled effect estimate comparing PTCs with ATCs for complications covered the null value (3 studies; OR, 0.80; 95% CI, 0.41-1.56) (eFigure 23 in Supplement 1). Results were similar when we restricted to studies with the same designation level in intervention and control groups (OR, 0.80; 95% CI, 0.41-1.57 for level I and II) (Table 2; eFigure 24 in Supplement 1). There were insufficient studies for other subgroup analyses.

### Processes of Care

Compared with ATCs, PTCs were associated with a reduction of 52% in the odds of CT use (7 studies; OR, 0.48; 95% CI, 0.26-0.89) and 64% in operative management for SOI (8 studies; OR, 0.36; 95% CI, 0.23-0.57) (Table 2; eFigure 25, and eFigure 26 in Supplement 1). However, heterogeneity was considerable (*I^2^* = 96% and *I^2^* = 76%, respectively) and prediction intervals included nonbeneficial effect estimates.

When we restricted to studies with the same designation level in intervention and control groups and to trauma centers verified by the same organization, results were similar for operative management of SOI (OR, 0.38; 95% CI, 0.21-0.67 for levels I and II; and OR, 0.24; 95% CI, 0.24-0.24 for centers verified by the same organization) (Table 2; eFigure 27, eFigure 28, and eFigure 29 in Supplement 1). There were insufficient studies for other subgroup analyses.

### PTCs vs CTCs

In the 11 studies comparing PTCs with CTCs for mortality, the 95% CI on the pooled OR was closer to the null value but was still statistically significant (OR, 0.73; 95% CI, 0.53-0.99) (Table 2; eFigure 30 in Supplement 1). There were insufficient studies for subgroup analyses.

### Results Not Included in Meta-Analyses

Studies not included in the meta-analyses suggested that PTCs may be associated with a more favorable discharge destination, lower resource use, fewer blood product transfusions, less interventional radiology, and fewer tracheostomies. Results are shown in eTable 6 in Supplement 1.

### Certainty of Evidence

Certainty of evidence was very low for all outcomes (Table 3). It was upgraded for mortality because of a dose-response association but then downgraded for possible risk of publication bias.

**Table 3.** Grading of Recommendations Assessment, Development and Evaluation of Evidence for Outcomes Included in Meta-Analyses

Outcomes (No. of studies)	Risk of bias^a^	Inconsistency	Indirectness	Imprecision	Publication bias^b^	Magnitude	Residual bias	Dose-response	Certainty of evidence
Mortality (25)	Serious	No	No	No	Yes	No	No	Yes^c^	Very low
Imaging (3)	Serious	No	No	No	NA	No	No	No	Very low
Surgery (7)	Serious	No	No	No	NA	No	No	No	Very low
Complications (5)	Critical	Yes	No	No	NA	No	No	No	Very low

^a^ Risk of bias is based on Risk of Bias in Nonrandomized Studies of Interventions tool with its 7 components (bias due to confounding, selection of study participants, classification of intervention, deviations from intended interventions, missing data, measurement of outcomes, and selection in the reported results).

^b^ Could only be evaluated for mortality.

^c^ Association was greater in younger than in older children and effect was greater for pediatric trauma centers vs adult trauma centers than for pediatric trauma centers vs combined adult and pediatric trauma centers.

## Discussion

The results of our systematic review and meta-analysis suggest that compared with ATCs, PTCs are associated with 40% lower odds of mortality, 50% lower odds of CT use, and 60% lower odds of operative SOI management for children admitted to hospitals following trauma. Statistical heterogeneity was considerable overall but was low to moderate when outliers were removed. Associations with mortality were closer to the null and not statistically significant for adolescents and for studies with critical risk of bias. Results for other subgroup and sensitivity analyses were similar to the main analysis. PTCs were associated with a 30% lower odds of mortality compared with CTCs. No association was observed between complications and trauma center type. Certainty of evidence was very low for all outcomes.

The reduction in mortality observed in our study was greater than that reported in a 2006 meta-analysis^5^ comparing mortality before and after the establishment of trauma systems in all age groups (15% reduction). However, we observed similar reductions in mortality to meta-analyses comparing level I trauma centers to other hospitals for adults with major trauma (33% reduction)^10^ or trauma centers vs nontrauma centers for all trauma admissions (36%).^9^ In this last meta-analysis, a subgroup analysis suggested 60% lower odds of mortality when children younger than 19 years were treated in a mature trauma system compared with a nontrauma or early-stage trauma system. Our findings are also in keeping with a large body of literature suggesting that adherence to guidelines and patient outcomes are better when children are treated at pediatric institutions, across a variety of conditions.^77,78^ This could be due to additional pediatric specialty training,^79^ better pediatric readiness and access to pediatric-specific equipment or interventions,^80^ or volume-outcome effects.^81^ The association for mortality comparing PTCs with CTCs supports these hypotheses. Our results also suggest that the advantage of PTCs over ATCs holds whatever the designation level or verification status.

We did not observe a statistically significant association between trauma center type and mortality for older adolescents, which suggests that the advantage of PTCs may not hold for this population. However, our results do not clarify what the optimal age cutoff should be or whether other factors such as pubertal status and size should be accounted for. Although the American Academy of Pediatrics discourages the use of arbitrary age limits to guide clinical care,^82^ the absence of age criteria in clinical decision rules, including triage tools^83^ and clinical practice guidelines,^84^ is a major barrier to their implementation.

Our results support the advantage of PTCs in the subpopulation of children with head injuries and penetrating injuries. Results on SOI were not statistically significant but also suggested an association with more favorable outcomes in PTCs than ATCs. Our observations may reflect the differences in treatment recommendations for these injuries between children and adults.^85,86^ We did not have sufficient data to evaluate associations for other types of injuries including rare pathologies such as vascular or urological injuries, for which expertise in PTCs may be lacking.^87,88^

Given the risks of exposure to ionizing radiation in children, multiple guidelines emit recommendations on limiting the use of CT in pediatric populations.^89,90,91^ Similarly, guidelines suggest operative management can be avoided in favor of interventional radiology or watchful waiting in higher-grade injuries in children than in adults.^86,92^ Our results suggest that PTCs have higher adherence to these guidelines than ATCs. However, we were unable to isolate inappropriate CT use or operative management, as this information was not reported in most studies. One of the studies included suggested that less aggressive approaches to SOIs among adolescents in PTCs may lead to higher mortality.^13^

### Limitations 

#### Limitations of Evidence

The risk of bias was serious or critical in all included studies, mostly due to confounding or selection bias. The improved outcomes observed in PTC could be explained by immortal time bias, whereby very sick children are not stable enough to transfer to PTCs. However, studies excluding or adjusting for transfers-in did not observe different results. Results could also be explained by a lower threshold for admission in PTCs or increased bed pressure in ATCs, but most studies did not include children with minor injuries and meta-analysis restricted to major trauma did not change our observations. Included studies based on trauma registries with facultative participation may be subject to selection bias, whereby hospitals with more quality improvement resources are more likely to participate. However, this would be expected to lead to an underestimation of the advantage of PTCs. Additionally, complications may be better recorded or more thoroughly screened in PTCs,^93^ which may explain the lack of benefit of PTCs for this outcome.

Other limits of the body of evidence include the lack of studies on outcomes related to functional status and quality of life, which may be more important to injured children and their families, and the lack of data on costs, which is critical to providing high-value and sustainable health care.^94^ Furthermore, we were unable to conduct subgroup analyses for outcomes other than mortality and low sample sizes led to very imprecise estimates of association in most subgroup analyses. Additionally, heterogeneity of our pooled estimates was high and was not reduced for subgroup analyses other than type of injury. There are therefore likely to be other factors explaining heterogeneity that could not be explored in this study.

#### Limitations of the Review Process

We had to rely on authors’ definitions of trauma center designation, which likely varied across systems. However, analyses limited to ACS-verified centers led to similar results. We were unable to limit our review to major trauma because studies used heterogeneous criteria to define injury severity (ISS, abbreviated injury scale, physiological criteria, length of stay, interventions). Along with heterogeneous age criteria, this likely reflects the lack of consensus on which patients should be sent to a PTC, highlighted by the imperfect accuracy of trauma triage tools, particularly in pediatric populations.^95^ Additionally, the evaluation of publication bias and its inclusion in grades of evidence is controversial^96^; without this criterion, the evidence on mortality would have been rated low rather than very low.

## Conclusions

The results of our study suggest that PTCs may be associated with better outcomes than ATCs or CTCs for children hospitalized following trauma. Results suggest there could be an age cutoff beyond which transfer to a PTC may not be beneficial, but current literature does not provide sufficient evidence on what that cutoff should be. Similarly, our results suggest that PTCs are associated with better outcomes than ATCs for children with head injuries and penetrating injuries, but we did not identify sufficient evidence to extend results to other types of injury. We recommend that future studies better address selection bias by conducting studies on samples that are population-based or have uniform and clearly justified inclusion or exclusion criteria. Confounding control could be improved by adequately adjusting for important confounders including physiological status on arrival and using state-of-the-art methods such as target trial emulation. Future research should also strive to further clarify which patients most benefit from definitive treatment in a PTC, to evaluate functional status and quality of life, and to study the cost-effectiveness of PTCs.

## Notes

Supplement 1. **eAppendix 1.** Methods **eTable 1.** Definitions Used for Study Inclusion Criteria **eTable 2.** Search Strategies **eTable 3.** Definitions Used for Study Outcomes **eTable 4.** Subgroup and Sensitivity Analyses **eFigure 1.** Preferred Reporting Items for Systematic Reviews and Meta-Analysis Flow Diagram **eAppendix 2.** Excluded Studies **eTable 5.** Characteristics of Included Studies **eAppendix 3.** Included Studies **eFigure 2.** Enhanced Funnel Plot of Studies Evaluating the Odds of mortality in Pediatric Major Trauma at Pediatric Trauma Centers vs Adult Trauma Centers (Trim and Fill Method) **eFigure 3.** Forest Plots Describing the Odds of Mortality for Children Aged 15 Years or Less Admitted to Hospitals Following Trauma Treated at Pediatric Trauma Centers (PTCs) Versus Adult Trauma Centers (ATCs) **eFigure 4.** Forest Plots Describing the Odds of Mortality for Children Aged 14 Years or Less Admitted to Hospitals Following Trauma Treated at pediatric Trauma Centers (PTCs) Versus Adult Trauma Centers (ATCs) **eFigure 5.** Forest Plots Describing the Odds of Mortality for Children With Head Injury Treated at Pediatric Trauma Centers (PTCs) vs Adult Trauma Centers (ATCs) **eFigure 6.** Forest Plots Describing the Odds of Mortality for Children With Blunt Solid Organ Injury Treated at Pediatric Trauma Centers (PTCs) vs Adult Trauma Centers (ATCs) **eFigure 7.** Forest Plots Describing the Odds of Mortality for Children With Penetrating Trauma Treated at Pediatric Trauma Centers (PTCs) vs Adult Trauma Centers (ATCs) **eFigure 8.** Forest Plots Describing the Odds of Mortality for Children With Major Trauma (ISS ≥ 12) Treated at Pediatric Trauma Centers (PTCs) vs Adult Trauma Centers (ATCs) **eFigure 9.** Forest Plots Describing the Odds of Mortality for children Admitted to Hospitals Following Trauma Treated at Level I Pediatric Trauma Centers (PTCs) vs Level I Adult Trauma Centers (ATCs) **eFigure 10.** Forest Plots Describing the Odds of Mortality for Children Admitted to Hospitals Following Trauma Treated at Level I and II Pediatric Trauma Centers (PTCs) vs Level I and II Adult Trauma Centers (ATCs) **eFigure 11.** Forest Plots Describing the Odds of Mortality for children Admitted to Hospitals Following Trauma Treated at Level I and II Pediatric Trauma Centers (PTCs) vs Level I and II Adult Trauma Centers (ATCs) With the Same Verification Organization **eFigure 12.** Forest Plots Describing the Odds of Mortality for Children Admitted to Hospitals Following Trauma Treated at Pediatric Trauma Centers (PTCs) vs Adult Trauma Centers (ATCs) in the US **eFigure 13.** Forest Plots Describing the Odds of Mortality for Children Admitted to Hospitals Following Trauma Treated at Pediatric Trauma Centers (PTCs) vs Adult Trauma Centers (ATCs) in Other Countries **eFigure 14.** Forest Plots Describing the Odds of Mortality for Children Admitted to Hospitals Following Trauma Treated at Pediatric Trauma Centers (PTCs) vs Adult Trauma Centers (ATCs) in Studies Recruiting Patients between 2010 to 2014 **eFigure 15.** Forest Plots Describing the Odds of Mortality for Children Admitted to Hospitals Following Trauma Treated at Pediatric Trauma Centers (PTCs) vs Adult Trauma Centers (ATCs) in Studies Recruiting Patients between 2015 to 2023 **eFigure 16.** Forest Plots Describing the Odds of Mortality for Children Admitted to Hospitals Following Trauma Treated at Pediatric Trauma Centers (PTCs) vs Adult Trauma Centers (ATCs) With Studies at Moderate (n=1) or Serious Risk of Bias **eFigure 17.** Forest Plots Describing the Odds of Mortality for Children Admitted to Hospitals Following Trauma Treated at Pediatric Trauma Centers (PTCs) vs Adult Trauma Centers (ATCs) With Studies at Critical Risk of Bias **eFigure 18.** Forest Plots Describing the Odds of Mortality for Children Admitted to Hospitals Following Trauma Treated at Pediatric Trauma Centers (PTCs) vs Adult Trauma Centers (ATCs) for Studies With Transfers Excluded **eFigure 19.** Forest Plots Describing the Odds of Mortality for Children Admitted to Hospitals Following Trauma Treated at Pediatric Trauma Centers (PTCs) vs Adult Trauma Centers (ATCs) for Studies With Transfers Included and Adjusted For **eFigure 20.** Forest Plots Describing the Odds of Mortality for Children Admitted to Hospitals Following Trauma Treated at Pediatric Trauma Centers (PTCs) vs Adult Trauma Centers (ATCs) for Studies With Transfers Included and Not Adjusted For **eFigure 21.** Forest Plots Describing the Odds of Mortality for Children Admitted to Hospitals Following Trauma Treated at Pediatric Trauma Centers (PTCs) vs Adult Trauma Centers (ATCs) With 7 Outliers Removed **eFigure 22****.** Forest Plots Describing the Odds of Mortality for Children Admitted to Hospitals Following Trauma Treated at Pediatric Trauma Centers (PTCs) vs Adult Trauma Centers (ATCs) With 3 Outliers Removed **eFigure 23.** Forest Plots Describing the Odds of Complications for Children Admitted to Hospitals Following Trauma Treated at pediatric Trauma Centers (PTCs) vs Adult Trauma Centers (ATCs) **eFigure 24.** Forest Plots Describing the Odds of Complications for Children Admitted to Hospitals Following Trauma Treated at Level I and II Pediatric Trauma Centers (PTCs) vs Level I and II Adult Trauma Centers (ATCs) **eFigure 25.** Forest Plots Describing the Odds of Computed Tomography (CT) Imaging for Children Admitted to Hospitals Following Trauma Treated at Pediatric Trauma Centers (PTCs) vs Adult Trauma Centers (ATCs) **eFigure 27.** Forest Plots Describing the Odds of Operative Management for Children Admitted to Hospitals Following Trauma Treated at Level I Pediatric Trauma Centers (PTCs) vs Level I Adult Trauma Centers (ATCs) **eFigure 26.** Forest Plots Describing the Odds of Operative Management for Children With Blunt Solid Organ Injuries Treated at Pediatric Trauma Centers (PTCs) vs Adult Trauma Centers (ATCs) **eFigure 28.** Forest Plots Describing the Odds of Operative Management for Children Admitted to Hospitals Following Trauma Treated at Level I and II Pediatric Trauma Centers (PTCs) vs Level I and II Adult Trauma Centers (ATCs) **eFigure 29.** Forest Plots Describing the Odds of Operative Management for Children Admitted to Hospitals Following Trauma Treated at Level I and II Pediatric Trauma Centers (PTCs) vs Level I and II Adult Trauma Centers (ATCs) With the Same Verification Organization **eFigure 30.** Forest Plots Describing the Odds of Mortality for Children Admitted to Hospitals Following Trauma Treated at Pediatric Trauma Centers (PTCs) vs Combined Adult and Pediatric Centers (CTCs) **eTable 6.** Results Not Included in Meta-Analyses

Supplement 2.
Data Sharing Statement
