# Supplementary material for: Pediatric vs Adult or Mixed Trauma Centers in Children Admitted to Hospitals Following Trauma: A Systematic Review and Meta-Analysis
Source: JAMA Netw Open. 2023 Sep 18;6(9):e2334266. doi: 10.1001/jamanetworkopen.2023.34266 (PMC10507486; doi:10.1001/jamanetworkopen.2023.34266)
Supplement: Supplement 1. — eAppendix 1. Methods eTable 1. Definitions Used for Study Inclusion Criteria eTable 2. Search Strategies eTable 3. Definitions Used for Study Outcomes eTable 4. Subgroup and Sensitivity Analyses eFigure 1. Preferred Reporting Items for Systematic Reviews and Meta-Analysis Flow Diagram eAppendix 2. Excluded Studies eTable 5. Characteristics of Included Studies eAppendix 3. Included Studies eFigure 2. Enhanced Funnel Plot of Studies Evaluating the Odds of mortality in Pediatric Major Trauma at Pediatric Trauma Centers vs Adult Trauma Centers (Trim and Fill Method) eFigure 3. Forest Plots Describing the Odds of Mortality for Children Aged 15 Years or Less Admitted to Hospitals Following Trauma Treated at Pediatric Trauma Centers (PTCs) Versus Adult Trauma Centers (ATCs) eFigure 4. Forest Plots Describing the Odds of Mortality for Children Aged 14 Years or Less Admitted to Hospitals Following Trauma Treated at pediatric Trauma Centers (PTCs) Versus Adult Trauma Centers (ATCs) eFigure 5. Forest Plots Describing the Odds of Mortality for Children With Head Injury Treated at Pediatric Trauma Centers (PTCs) vs Adult Trauma Centers (ATCs) eFigure 6. Forest Plots Describing the Odds of Mortality for Children With Blunt Solid Organ Injury Treated at Pediatric Trauma Centers (PTCs) vs Adult Trauma Centers (ATCs) eFigure 7. Forest Plots Describing the Odds of Mortality for Children With Penetrating Trauma Treated at Pediatric Trauma Centers (PTCs) vs Adult Trauma Centers (ATCs) eFigure 8. Forest Plots Describing the Odds of Mortality for Children With Major Trauma (ISS ≥ 12) Treated at Pediatric Trauma Centers (PTCs) vs Adult Trauma Centers (ATCs) eFigure 9. Forest Plots Describing the Odds of Mortality for children Admitted to Hospitals Following Trauma Treated at Level I Pediatric Trauma Centers (PTCs) vs Level I Adult Trauma Centers (ATCs) eFigure 10. Forest Plots Describing the Odds of Mortality for Children Admitted to Hospitals Following Trauma Treated at Level I and II Pe [file jamanetwopen-e2334266-s001.pdf]

Supplemental Online Content

Moore L, Freire G, Turgeon A, et al. Pediatric vs adult or mixed trauma centers in children admitted to hospitals following trauma: a systematic review and meta-analysis. *JAMA Netw Open*. 2023;6(9):e2334266. doi:10.1001/jamanetworkopen.2023.34266

eTable 1. Definitions Used for Study Inclusion Criteria

eTable 2. Search Strategies

eTable 3. Definitions Used for Study Outcomes

eTable 4. Subgroup and Sensitivity Analyses

eReferences 1. References From eAppendix 1

eFigure 1. Preferred Reporting Items for Systematic Reviews and Meta-Analysis Flow Diagram

eReferences 2. References of Excluded Studies

eTable 5. Characteristics of Included Studies

eReferences 3. References of Included Studies

eFigure 2. Enhanced Funnel Plot of Studies Evaluating the Odds of mortality in Pediatric Major Trauma at Pediatric Trauma Centers vs Adult Trauma Centers (Trim and Fill Method)

eFigure 3. Forest Plots Describing the Odds of Mortality for Children Aged 15 Years or Less Admitted to Hospitals Following Trauma Treated at Pediatric Trauma Centers (PTCs) Versus Adult Trauma Centers (ATCs)

eFigure 4. Forest Plots Describing the Odds of Mortality for Children Aged 14 Years or Less Admitted to Hospitals Following Trauma Treated at pediatric Trauma Centers (PTCs) Versus Adult Trauma Centers (ATCs)

eFigure 5. Forest Plots Describing the Odds of Mortality for Children With Head Injury Treated at Pediatric Trauma Centers (PTCs) vs Adult Trauma Centers (ATCs)

eFigure 6. Forest Plots Describing the Odds of Mortality for Children With Blunt Solid Organ Injury Treated at Pediatric Trauma Centers (PTCs) vs Adult Trauma Centers (ATCs)

eFigure 7. Forest Plots Describing the Odds of Mortality for Children With Penetrating Trauma Treated at Pediatric Trauma Centers (PTCs) vs Adult Trauma Centers (ATCs)

eFigure 8. Forest Plots Describing the Odds of Mortality for Children With Major Trauma (ISS ≥ 12) Treated at Pediatric Trauma Centers (PTCs) vs Adult Trauma Centers (ATCs)

© 2023 Moore L et al. *JAMA Network Open.*

eFigure 9. Forest Plots Describing the Odds of Mortality for children Admitted to Hospitals Following Trauma Treated at Level I Pediatric Trauma Centers (PTCs) vs Level I Adult Trauma Centers (ATCs)

eFigure 10. Forest Plots Describing the Odds of Mortality for Children Admitted to Hospitals Following Trauma Treated at Level I and II Pediatric Trauma Centers (PTCs) vs Level I and II Adult Trauma Centers (ATCs)

eFigure 11. Forest Plots Describing the Odds of Mortality for children Admitted to Hospitals Following Trauma Treated at Level I and II Pediatric Trauma Centers (PTCs) vs Level I and II Adult Trauma Centers (ATCs) With the Same Verification Organization

eFigure 12. Forest Plots Describing the Odds of Mortality for Children Admitted to Hospitals Following Trauma Treated at Pediatric Trauma Centers (PTCs) vs Adult Trauma Centers (ATCs) in the US

eFigure 13. Forest Plots Describing the Odds of Mortality for Children Admitted to Hospitals Following Trauma Treated at Pediatric Trauma Centers (PTCs) vs Adult Trauma Centers (ATCs) in Other Countries

eFigure 14. Forest Plots Describing the Odds of Mortality for Children Admitted to Hospitals Following Trauma Treated at Pediatric Trauma Centers (PTCs) vs Adult Trauma Centers (ATCs) in Studies Recruiting Patients up to 2010 to 2014

eFigure 15. Forest Plots Describing the Odds of Mortality for Children Admitted to Hospitals Following Trauma Treated at Pediatric Trauma Centers (PTCs) vs Adult Trauma Centers (ATCs) in Studies Recruiting Patients up to 2015 to 2023

eFigure 16. Forest Plots Describing the Odds of Mortality for Children Admitted to Hospitals Following Trauma Treated at Pediatric Trauma Centers (PTCs) vs Adult Trauma Centers (ATCs) With Studies at Moderate (n=1) or Serious Risk of Bias

eFigure 17. Forest Plots Describing the Odds of Mortality for Children Admitted to Hospitals Following Trauma Treated at Pediatric Trauma Centers (PTCs) vs Adult Trauma Centers (ATCs) With Studies at Critical Risk of Bias

eFigure 18. Forest Plots Describing the Odds of Mortality for Children Admitted to Hospitals Following Trauma Treated at Pediatric Trauma Centers (PTCs) vs Adult Trauma Centers (ATCs) for Studies With Transfers Excluded

eFigure 19. Forest Plots Describing the Odds of Mortality for Children Admitted to Hospitals Following Trauma Treated at Pediatric Trauma Centers (PTCs) vs Adult Trauma Centers (ATCs) for Studies With Transfers Included and Adjusted For

eFigure 20. Forest Plots Describing the Odds of Mortality for Children Admitted to Hospitals Following Trauma Treated at Pediatric Trauma Centers (PTCs) vs Adult Trauma Centers (ATCs) for Studies With Transfers Included and Not Adjusted For

eFigure 21. Forest Plots Describing the Odds of Mortality for Children Admitted to Hospitals Following Trauma Treated at Pediatric Trauma Centers (PTCs) vs Adult Trauma Centers (ATCs) With 7 Outliers Removede

© 2023 Moore L et al. *JAMA Network Open.*

Figure 22. Forest Plots Describing the Odds of Mortality for Children Admitted to Hospitals Following Trauma Treated at Pediatric Trauma Centers (PTCs) vs Adult Trauma Centers (ATCs) With 3 Outliers Removed

eFigure 23. Forest Plots Describing the Odds of Complications for Children Admitted to Hospitals Following Trauma Treated at pediatric Trauma Centers (PTCs) vs Adult Trauma Centers (ATCs)

eFigure 24. Forest Plots Describing the Odds of Complications for Children Admitted to Hospitals Following Trauma Treated at Level I and II Pediatric Trauma Centers (PTCs) vs Level I and II Adult Trauma Centers (ATCs)

eFigure 25. Forest Plots Describing the Odds of Computed Tomography (CT) Imaging for Children Admitted to Hospitals Following Trauma Treated at Pediatric Trauma Centers (PTCs) vs Adult Trauma Centers (ATCs)

eFigure 27. Forest Plots Describing the Odds of Operative Management for Children Admitted to Hospitals Following Trauma Treated at Level I Pediatric Trauma Centers (PTCs) vs Level I Adult Trauma Centers (ATCs)

eFigure 26. Forest Plots Describing the Odds of Operative Management for Children With Blunt Solid Organ Injuries Treated at Pediatric Trauma Centers (PTCs) vs Adult Trauma Centers (ATCs)

eFigure 28. Forest Plots Describing the Odds of Operative Management for Children Admitted to Hospitals Following Trauma Treated at Level I and II Pediatric Trauma Centers (PTCs) vs Level I and II Adult Trauma Centers (ATCs)

eFigure 29. Forest Plots Describing the Odds of Operative Management for Children Admitted to Hospitals Following Trauma Treated at Level I and II Pediatric Trauma Centers (PTCs) vs Level I and II Adult Trauma Centers (ATCs) With the Same Verification Organization

eFigure 30. Forest Plots Describing the Odds of Mortality for Children Admitted to Hospitals Following Trauma Treated at Pediatric Trauma Centers (PTCs) vs Combined Adult and Pediatric Centers (CTCs)

eTable 6. Results Not Included in Meta-Analyses

This supplemental material has been provided by the authors to give readers additional information about their work.

© 2023 Moore L et al. *JAMA Network Open.*

eAppendix 1. Methods

Eligibility criteria

Eligible studies were prospective or retrospective cohort studies, case-control studies or case-cohort studies that compared outcomes of children (≤ 19 years of age) admitted to hospitals following trauma in PTCs to those in ATCs, CTCs, or non-designated hospitals (eTable 1).

eTable 1. Definitions Used for Study Inclusion Criteria

**Inclusion criteria**	
Pediatric patients	Children or adolescents ≤19 years of age[1,2]
Hospital admission	Admitted to an acute care hospital – admission to the ward or died in the emergency department
PTC	Acute care hospital equipped and staffed to provide care for **pediatric** patients[1] suffering from major traumatic injuries, designated by a recognised authority (e.g. state, American College of Surgeons)
ATC	Acute care hospital equipped and staffed to provide care for **adult** patients[1] suffering from major traumatic injuries, designated by a recognised authority (e.g. state, American College of Surgeons)
CTC	Acute care hospital equipped and staffed to provide care for **adult or pediatric** patients[1] suffering from major traumatic injuries, designated by a recognised authority (e.g. state, American College of Surgeons)

PTC, pediatric trauma center; ATC: adult trauma center; CTC, combined trauma center

[1]There is currently no consensus on age cut-offs for pediatric trauma populations so the World Health Organisation definition of children and adolescents was used (https://www.who.int/health-topics/adolescent-health#tab=tab_1)

[2]Studies including patients >19 years of age were included if ≤ 19-year-olds represented more than 80% of the study population.

We applied no restrictions to language or date of publication. Articles in languages other than English were translated. If information to be extracted was unavailable or unclear, we contacted the study authors for clarification. Studies reporting on diverse populations were included if data on pediatric trauma patients could be extracted. We also excluded studies presenting no data on selected outcomes and exposures, conference abstracts, unpublished studies, narrative reviews, case reports, in addition to studies reporting drowning, burns, foreign bodies, poisoning, late effects of injury, cadaver or animal studies.

Information sources

We conducted a systematic search of the Medical Literature Analysis and Retrieval System Online (MEDLINE, via PubMed), Excerpta Medica dataBASE (EMBASE), Web of Science and Cumulative Index to Nursing and Allied Health Literature (CINAHL) from inception to up to February 28th, 2023. Thesis repositories, healthcare quality websites and references of included studies were also screened.

Search strategy

Our search strategy was designed using a combination of Boolean terms with relevant keywords and subject headings for EMBASE (EMBASE tree; EMTREE) and MEDLINE (Medical Subject Headings; MeSH), and then adapted to the remaining databases (eTable 2). Clinicians practising in pediatric trauma care and information specialists were consulted to refine the search strategy using the Peer Review of Electronic Search Strategies checklist.[1]

eTable 2. Search Strategies

Concepts	PubMed search strategy	Search	# Results
Pediatric trauma	("Wounds and Injuries"[Mesh:NoExp] OR Trauma*[TIAB]) AND (child[Mesh:NoExp] OR child*) OR "Pediatric Emergency Medicine"[Mesh]	#1	90,491
Trauma center	"Trauma Centers"[Mesh] OR pediatric trauma cent*[TIAB] OR Adult trauma cent*[TIAB]	#2	12,990
Total for PubMed	#1 AND #2	#3	2,792
**Concepts**	**Embase search strategy**	**Search**	**# Results**

© 2023 Moore L et al. *JAMA Network Open.*

Pediatric trauma	'injury'/de AND 'child'/de OR 'pediatric emergency medicine'/de	#4	36,323
Trauma center	'emergency health service'/de OR "pediatric trauma cent*":ti,ab,kw OR "Adult trauma cent*":ti,ab,kw	#5	113,274
Total for Embase	#4 OR #5	#6	1,511
**Concepts**	**Web of Science search strategy**	**Search**	**# Results**
Pediatric trauma	TS=(injury or child*)	#7	3,233,494
Trauma center	TS=("pediatric trauma cent*" OR "Adult trauma cent*")	#8	1,139
Total for Web of Science	#7 OR #8	#9	1,049
**Concepts**	**CINAHL search strategy**	**Search**	**# Results**
Pediatric trauma	MH Child AND MH "Wounds and Injuries"	#10	5,456
Trauma center	MH "Trauma Centers" OR TI "pediatric trauma cent*" OR AB "pediatric trauma cent*" OR TI Adult trauma cent* OR AB Adult trauma cent*	#11	3,142
Total for CINAHL	Limit to articles since 2007	#12	200

Selection process
Pairs of reviewers (KM, LM, PAT, AA, TD) independently assessed study eligibility. Inter-reviewer agreement on eligibility was assessed using the first 500 citations. We repeated this process until acceptable inter-rater agreement was attained. Discrepancies between reviewers were resolved by consensus and a third reviewer adjudicated when necessary (LM). Citations were managed using EndNote software (version X9.3.3, New York City: Thomson Reuters, 2018). We managed duplicates via electronic and manual screening. If multiple publications based on the same dataset are identified by crosschecking authors, dates and settings, we selected only one publication for analyses based on study dates (most recent) and sample size (largest).

Data collection process
We developed a standard electronic data extraction form and a detailed instruction manual and piloted it on a representative sample of ten publications. Pairs of reviewers (KM, LM, PAT) independently extracted data including study design, setting (country), patient characteristics (age, injury mechanisms, type and severity of injuries), hospital characteristics (PTC/ATC/CTC, trauma center designation level, and verification organisation), outcome measures, adjustment variables, and measures of association and variation.

Outcomes
Our primary outcomes of interest, defined *a priori* in collaboration with our advisory committee, were mortality, complications, functional status, discharge destination, and quality of life (eTable 3). Secondary outcomes were resource utilisation and processes of care. For example, use of computed tomography [CT] imaging or operative management of blunt solid organ injuries.

eTable 3. Definitions Used for Study Outcomes

**Outcomes**	
Mortality	Any emergency department or in-patient death, all causes
Complications	In-patient complications as defined by authors[1]
Functional status	As measured by a validated instrument (e.g. Glasgow Outcome Scale, the Functional Independence Measure) on discharge or during follow-up
Discharge destination	Patient destination on hospital discharge (e.g. home, rehabilitation, long-term care)
Resource use	Hospital length of stay, intensive care unit admission, intensive care unit length of stay, ventilator days, costs, or charges
Processes of care	Any diagnostic or therapeutic intervention considered by the authors as an outcome of interest (e.g., use of computed

© 2023 Moore L et al. *JAMA Network Open.*

	tomography imaging or operative management of blunt solid organ injuries[2])

[1]Currently no consensus on a definition of complications for pediatric injury populations
[2]Does not include interventional radiology

Risk of bias assessment
Three content experts (LM, PAT, JG) independently rated risk of bias using the Risk of Bias in Non-randomized Studies of Interventions (ROBINS-I) tool.[2] The tool considers bias due to confounding, selection of participants into the study, misclassification of interventions, deviations from intended interventions, missing data, measurement of outcomes, and selection of the reported results. We resolved disagreements via arbitration with a third reviewer (NY). Two content experts (LM, PAT) evaluated the quality of evidence using Grading of Recommendations, Assessment, Development and Evaluation (GRADE) criteria.[3] We assessed publication bias using a contour-enhanced funnel plot and estimated the magnitude of the potential bias with the trim-and-fill method.[4]

Effect measures
We summarized data for dichotomous outcomes using odds ratios (ORs) along with 95% confidence intervals (CIs). Mean differences (MDs) or geometric mean ratios (GMRs) were used for continuous outcomes.

Synthesis methods
We restricted data synthesis to studies presenting risk-adjusted measures of association, i.e. measures minimally adjusted for age and injury severity; in the context of a review limited to observational studies with a very high risk of indication bias, unadjusted comparisons were considered not to produce meaningful results. When two studies or more evaluated the same exposure-outcome association, we conducted meta-analyses. We calculated pooled effect estimates and 95% confidence intervals (CIs) using random-effects models with an exact Mantel-Haenszel method, as design-related heterogeneity was present. Due to variations in sample sizes, we estimated between-study variance (τ^2) with the restricted maximum likelihood estimator and CIs with the Q-profile method. We applied a Hartung-Knapp correction in order to reflect uncertainty in estimating τ^2. We presented results using forest plots. We measured the heterogeneity of included studies using the I^2 statistic and interpreted as low if 0 to 40%, moderate if 30 to 60%, substantial if 50 to 90% and considerable if 75 to 100%. Given that I^2 tends toward 100% as sample sizes increase, we calculated prediction intervals around the pooled effect size as recommended in the Cochrane handbook.[5] These intervals present the range within which we expect the effect sizes of future studies when taking into consideration the current evidence. All analyses were conducted using R Statistical Software (4.2.1; R Core Team 2021) using the following R packages: meta (v6.0.0; Balduzzi, Rücker, and Schwarzer 2019), metafor (v3.8.1; Viechtbauer) and dmetar (Harrer, Cuijpers, Furukawa, T.A & Ebert, 2019).

Grading evidence
Two content experts (LM, PAT) independently graded evidence by applying Grading of Recommendations Assessment, Development and Evaluation (GRADE) criteria:[3] 1) risk of bias in the individual studies, 2) inconsistency, 3) indirectness, 4) imprecision, and 5) publication bias. In accordance with GRADE guidelines for grading evidence from non-randomized intervention studies, we then graded the certainty of evidence as high, moderate, low, or very low.

Pre-specified subgroup and sensitivity analyses
We conducted subgroup analyses for factors thought to modify the effectiveness of PTCs identified on consultation with our advisory committee: age, type of injury, injury severity, trauma center designation levels and verification body, country, and study period (eTable 4). To assess the impact of risk of bias on effect estimates, we repeated analyses excluding studies with a critical risk of bias. Finally, outliers were identified using a two-step analysis aiming to identify (1) studies whose upper bound of the 95% CI was lower than the lower bound of the pooled effect (extremely small effects) and (2) studies whose lower bound of the 95% CI was higher than the upper bound of the pooled effect (extremely large effects).[6]

© 2023 Moore L et al. *JAMA Network Open.*

eTable 4. Subgroup and Sensitivity Analyses

**Pre-specified**	
Age	≤15 years; 14-19 years[1]
Injury type	Traumatic brain; blunt solid organ; penetrating[2]
Injury severity	Major trauma (ISS≥12)[3]
Trauma center designation level	All level I; all level I or II ACS
Accrediting body	All TCs accredited by the same organisation (e.g. ACS)
Country	USA; other countries[4]
Study period	2010-2014; 2015-2019
Risk of bias	Moderate or serious[5]; critical
Outliers	According to recommendations
**Post-hoc**	
Patient transfers[6]	Excluded; included and adjusted for; included and not adjusted for

ISS, Injury Severity Score; ACS, American College of Surgeons; TC, trauma center; USA: United States of America
[1]Due to insufficient number of studies, preplanned categories 0-8 and 9-15 had to be grouped and preplanned category ≥16 had to be extended to 14 (11 studies focussed on adolescents but only one used an age cut-off of 16)
[2]Insufficient number of studies to conduct analyses for a priori categories spinal cord injury and major orthopedic injury
[3]Insifficient number of studies to conduct analyses for other categories of injury severity
[4]Insufficient number of studies to look at countries other than the US individually
[5]Only one study was moderate and none were low
[6]This subgroup analysis was added to assess the impact of what was considered potentially the most important source of bias

Protocol deviations

We were unable to limit our review to major trauma because studies used heterogeneous criteria to define injury severity (ISS, AIS, physiological criteria, LOS, interventions). We thus used ‘admission to an acute care hospital following injury’ as our inclusion criteria and conducted subgroup analysis for major trauma. We had to group pre-specified subgroup categories, <8 and 9-15 years of age due to the lack of studies on children 9-15 years. We had to extend our pre-planned threshold for older adolescents from 16 to 14 because although 11 studies focussed on adolescents, only one used an age cut-off of 16 years of age or greater. Planned subgroup analyses for country/continent had to be modified to analysis of studies conducted in the USA as only 19% of included studies were conducted in other countries. Due to lack of studies or differences in measures reported, we were unable to conduct meta-analyses for the following preplanned outcomes: functional status, quality of life, discharge destination, hospital and ICU LOS, ventilator days, and costs. We were also unable to conduct meta-analyses for the contrast PTC versus non-designated hospitals, and subgroup analyses for outcomes other than mortality and operative management.

eReferences 1. References From eAppendix 1

1. McGowan J, Sampson M, Salzwedel DM, et al. PRESS Peer Review of Electronic Search Strategies: 2015 Guideline Statement. *J Clin Epidemiol* 2016;75:40-6.
2. Sterne JA, Hernan MA, Reeves BC, et al. ROBINS-I: a tool for assessing risk of bias in non-randomised studies of interventions. *BMJ* 2016;355:i4919.
3. Brozek JL, Akl EA, Alonso-Coello P, et al. Grading quality of evidence and strength of recommendations in clinical practice guidelines. Part 1 of 3. An overview of the GRADE approach and grading quality of evidence about interventions. *Allergy* 2009;64:669-77.
4. Duval S, Tweedie R. Trim and fill: A simple funnel-plot-based method of testing and adjusting for publication bias in meta-analysis. *Biometrics* 2000;56:455-63.
5. Higgins J, Thomas J, Chandler J, et al. Cochrane Handbook for Systematic Reviews of Interventions version 6.3 [updated February 2022, Cochrane website]. Cochrane, 2022. Available at: www.training.cochrane.org/handbook. Accessed February 17th, 2023.
6. Harrer M, Cuijpers P, Furukawa TA, et al. Doing Meta-Analysis With R: A Hands-On Guide. 1st ed. Boca Raton, FL and London: Chapman & Hall/CRC Press; 2021.

© 2023 Moore L et al. *JAMA Network Open.*

eAppendix 2. Results (Tables, Figures and References)

eFigure 1. Preferred Reporting Items for Systematic Reviews and Meta-Analysis Flow Diagram

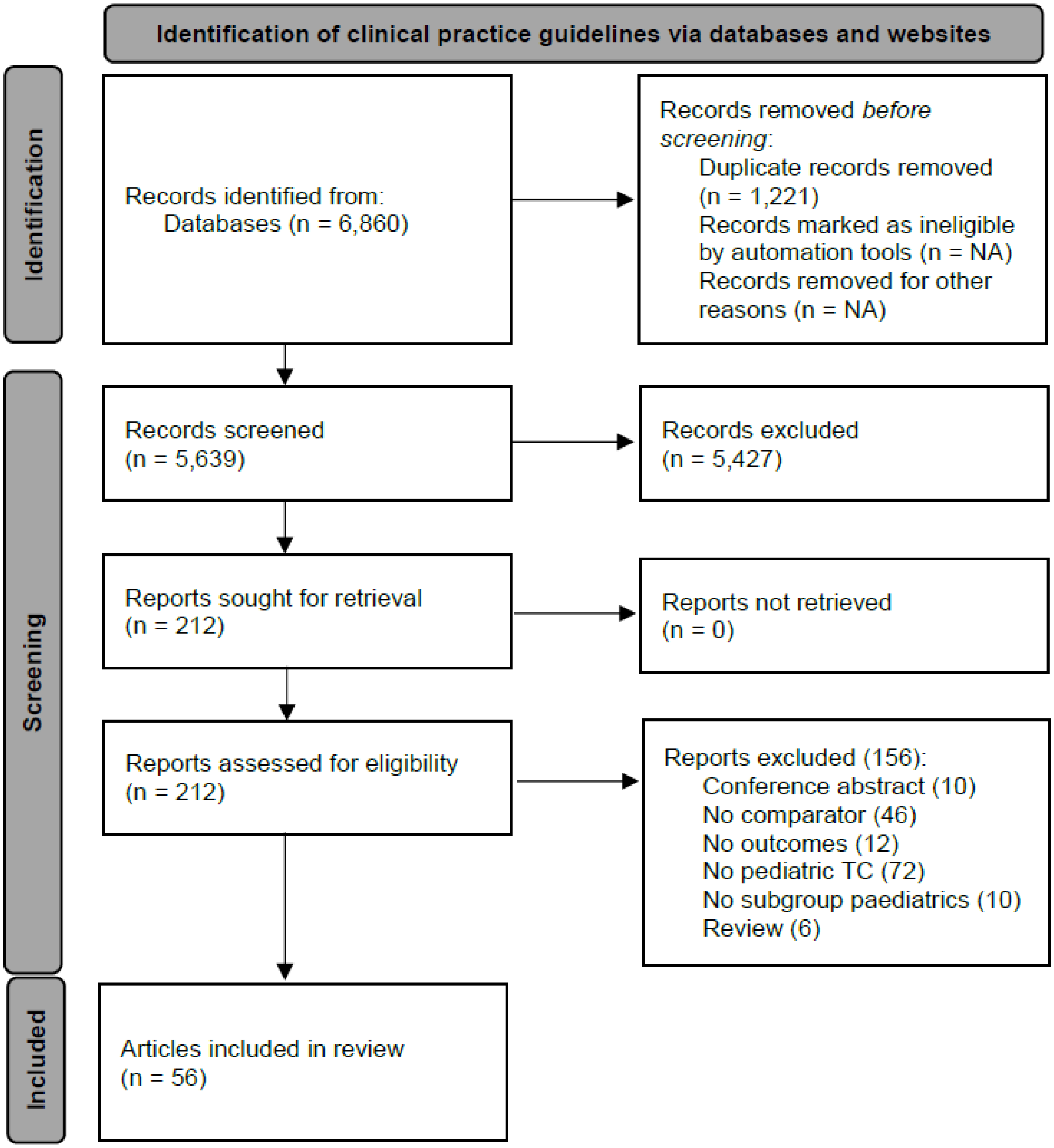

© 2023 Moore L et al. *JAMA Network Open.*

eReferences 2 – References of Excluded Studies

1. Cywes S, Rode H, Millar AJ. Blunt liver trauma in children: nonoperative management. *J Pediatr Surg* 1985;20:14-8.
2. Haller Jr JA, Buck J. Does a trauma-management system improve outcome for children with life-threatening injuries? *Canadian Journal of Surgery* 1985;28:477.
3. Eichelberger MR, Mangubat EA, Sacco WJ, Bowman LM, Lowenstein AD. Outcome analysis of blunt injury in children. *J Trauma* 1988;28:1109-17.
4. Smith JS, Jr., Martin LF, Young WW, Macioce DP. Do trauma centers improve outcome over non-trauma centers: the evaluation of regional trauma care using discharge abstract data and patient management categories. *J Trauma* 1990;30:1533-8.
5. Kaufmann CR, Maier RV, Kaufmann EJ, Rivara FP, Carrico CJ. Validity of applying adult TRISS analysis to injured children. *J Trauma* 1991;31:691-7; discussion 7-8.
6. Voeller GR, Mangiante EC, Fabian TC. The effect of a trauma system on the outcome of patients with pancreatic trauma. *Arch Surg* 1991;126:578-80.
7. Cooper A, Barlow B, Davidson L, Relethford J, O'Meara J, Mottley L. Epidemiology of pediatric trauma: importance of population-based statistics. *J Pediatr Surg* 1992;27:149-53; discussion 53-4.
8. Fortune JB, Sanchez J, Graca L, Haselbarth J, Kuehler DH, Wallace JR, et al. A pediatric trauma center without a pediatric surgeon: a four-year outcome analysis. *J Trauma* 1992;33:130-7; discussion 7-9.
9. Knudson MM, Shagoury C, Lewis FR. Can adult trauma surgeons care for injured children? *J Trauma* 1992;32:729-37; discussion 37-9.
10. Nakayama DK, Copes WS, Sacco W. Differences in trauma care among pediatric and nonpediatric trauma centers. *J Pediatr Surg* 1992;27:427-31.
11. Cooper A, Barlow B, DiScala C, String D, Ray K, Mottley L. Efficacy of pediatric trauma care: results of a population-based study. *J Pediatr Surg* 1993;28:299-303; discussion 4-5.
12. Mock CN, Adzotor KE, Conklin E, Denno DM, Jurkovich GJ. Trauma outcomes in the rural developing world: comparison with an urban level I trauma center. *J Trauma* 1993;35:518-23.
13. Rhodes M, Smith S, Boorse D. PEDIATRIC TRAUMA PATIENTS IN AN ADULT TRAUMA CENTER. *Journal of Trauma-Injury Infection and Critical Care* 1993;35:384-93.
14. Bensard DD, McIntyre RC, Jr., Moore EE, Moore FA. A critical analysis of acutely injured children managed in an adult level I trauma center. *J Pediatr Surg* 1994;29:11-8.
15. Mullins RJ, Veum-Stone J, Helfand M, Zimmer-Gembeck M, Hedges JR, Southard PA, et al. Outcome of hospitalized injured patients after institution of a trauma system in an urban area. *Journal of the American Medical Association* 1994;271:1919-24.
16. Rutledge R, Fakhry SM, Baker CC, Weaver N, Ramenofsky M, Sheldon GF, et al. A population-based study of the association of medical manpower with county trauma death rates in the United States. *Ann Surg* 1994;219:547-63; discussion 63-7.
17. Sola JE, Scherer LR, Haller JA, Jr., Colombani PM, Papa PA, Paidas CN. Criteria for safe cost-effective pediatric trauma triage: prehospital evaluation and distribution of injured children. *J Pediatr Surg* 1994;29:738-41.
18. D'Amelio LF, Hammond JS, Thomasseau J, Sutyak JP. "Adult" trauma surgeons with pediatric commitment: a logical solution to the pediatric trauma manpower problem. *Am Surg* 1995;61:968-74.
19. Keller MS, Vane DW. Management of pediatric blunt splenic injury: comparison of pediatric and adult trauma surgeons. *J Pediatr Surg* 1995;30:221-4; discussion 4-5.
20. Vane DW, Shackford SR. Epidemiology of rural traumatic death in children: a population-based study. *J Trauma* 1995;38:867-70.
21. Goldfarb MG, Bazzoli GJ, Coffey RM. Trauma systems and the costs of trauma care. *Health Serv Res* 1996;31:71-95.
22. Hall JR, Reyes HM, Meller JL, Loeff DS, Dembek R. The outcome for children with blunt trauma is best at a pediatric trauma center. *Journal of Pediatric Surgery* 1996;31:72-7.
23. Johnson DL, Krishnamurthy S. Send severely head-injured children to a pediatric trauma center. *Pediatric Neurosurgery* 1996;25:309-14.

© 2023 Moore L et al. *JAMA Network Open.*

24. Bernardo LM, Gardner MJ, Miracle TB, Henker R. Analgesic prescription and administration to adolescents admitted to an adult versus pediatric trauma center. *Journal of trauma nursing : the official journal of the Society of Trauma Nurses* 1997;4:13-21.
25. Hedges JR, Mann NC, Mullins RJ, Rowland D, Worrall W, Zechnich AD. Impact of a statewide trauma system on rural emergency department patient assessment documentation. OHSU Rural Trauma Research Group. *Acad Emerg Med* 1997;4:268-76.
26. Helling TS, Morse G, McNabney WK, Beggs CW, Behrends SH, HuttonRotert K, et al. Treatment of liver injuries at level I and level II centers in a multi-institutional metropolitan trauma system. *Journal of Trauma-Injury Infection and Critical Care* 1997;42:1091-6.
27. Hulka F, Mullins RJ, Mann NC, Hedges JR, Rowland D, Worrall WH, et al. Influence of a statewide trauma system on pediatric hospitalization and outcome. *J Trauma* 1997;42:514-9.
28. Mooney DP, Birkmeyer NJ, Udell JV, Shorter NA. Variation in the management of pediatric splenic injuries in New Hampshire. *J Pediatr Surg* 1998;33:1076-8; discussion 9-80.
29. Hulka F. Pediatric trauma systems: critical distinctions. *J Trauma* 1999;47:S85-9.
30. Konstantakos AK, Barnoski AL, Plaisier BR, Yowler CJ, Fallon WF, Jr., Malangoni MA. Optimizing the management of blunt splenic injury in adults and children. *Surgery* 1999;126:805-12; discussion 12-3.
31. Mangus RS, Mann NC, Worrall W, Mullins RJ. Statewide variation in the treatment of patients hospitalized with spleen injury. *Arch Surg* 1999;134:1378-84.
32. Mullins RJ, Mann NC. Introduction to the Academic Symposium to Evaluate Evidence Regarding the Efficacy of Trauma Systems. *J Trauma* 1999;47:S3-7.
33. Mullins RJ, Mann NC. Population-based research assessing the effectiveness of trauma systems. *J Trauma* 1999;47:S59-66.
34. Frumiento C, Vane DW. Changing patterns of treatment for blunt splenic injuries: an 11-year experience in a rural state. *J Pediatr Surg* 2000;35:985-8; discussion 8-9.
35. Partrick DA, Moore EE, Bensard DD, Karrer FM. Operative management of injured children at an adult level I trauma center. *J Trauma* 2000;48:894-901.
36. Potoka DA, Schall LC, Gardner MJ, Stafford PW, Peitzman AB, Ford HR. Impact of pediatric trauma centers on mortality in a statewide system. *Journal of Trauma-Injury Infection and Critical Care* 2000;49:237-45.
37. Athey J, Dean JM, Ball J, Wiebe R, Melese-d'Hospital I. Ability of hospitals to care for pediatric emergency patients. *Pediatr Emerg Care* 2001;17:170-4.
38. Clancy TV, Gary Maxwell J, Covington DL, Brinker CC, Blackman D. A statewide analysis of level I and II trauma centers for patients with major injuries. *J Trauma* 2001;51:346-51.
39. Jacobs IA, Kelly K, Valenziano C, Pawar J, Jones C. Nonoperative management of blunt splenic and hepatic trauma in the pediatric population: significant differences between adult and pediatric surgeons? *Am Surg* 2001;67:149-54.
40. Potoka D. Better outcomes for injured children - Pediatric trauma centers show lower mortality rates. *American Journal of Nursing* 2001;101:19-.
41. Potoka DA, Schall LC, Ford HR. Improved functional outcome for severely injured children treated at pediatric trauma centers. *Journal of Trauma-Injury Infection and Critical Care* 2001;51:824-32.
42. Sanchez JL, Lucas J, Feustel PJ. Outcome of adolescent trauma admitted to an adult surgical intensive care unit versus a pediatric intensive care unit. *Journal of Trauma - Injury, Infection and Critical Care* 2001;51:478-80.
43. Sherman HF, Landry VL, Jones LM. Should Level I trauma centers be rated NC-17? *J Trauma* 2001;50:784-91.
44. Wright JL, Klein BL. Regionalized pediatric trauma systems. *Clinical Pediatric Emergency Medicine* 2001;2:3-12.
45. Morrison W, Wright JL, Paidas CN. Pediatric trauma systems. *Crit Care Med* 2002;30:S448-56.
46. Simons R, Kasic S, Kirkpatrick A, Vertesi L, Phang T, Appleton L. Relative importance of designation and accreditation of trauma centers during evolution of a regional trauma system. *J Trauma* 2002;52:827-33; discussion 33-4.
47. Christensen EF, Laustrup TK, Høyer CC, Hougaard K, Spangsberg NL. [Mortality among severely injured patients before and after establishment of a trauma center in Aarhus]. *Ugeskr Laeger* 2003;165:4296-9.

© 2023 Moore L et al. *JAMA Network Open.*

48. Segui-Gomez M, Chang DC, Paidas CN, Jurkovich GJ, Mackenzie EJ, Rivara FP. Pediatric trauma care: an overview of pediatric trauma systems and their practices in 18 US states. *J Pediatr Surg* 2003;38:1162-9.
49. Barnett SJ, Saltzman DA. Efficacy of pediatric-specific trauma centers. *Pediatric critical care medicine : a journal of the Society of Critical Care Medicine and the World Federation of Pediatric Intensive and Critical Care Societies* 2004;5:93-4.
50. Farrell LS, Hannan EL, Cooper A. Severity of injury and mortality associated with pediatric blunt injuries: hospitals with pediatric intensive care units versus other hospitals. *Pediatr Crit Care Med* 2004;5:5-9.
51. Mooney DP, Forbes PW. Variation in the management of pediatric splenic injuries in New England. *J Trauma* 2004;56:328-33.
52. Todd SR, Arthur M, Newgard C, Hedges JR, Mullins RJ. Hospital factors associated with splenectomy for splenic injury: a national perspective. *J Trauma* 2004;57:1065-71.
53. Bowman SM, Zimmerman FJ, Christakis DA, Sharar SR, Martin DP. Hospital characteristics associated with the management of pediatric splenic injuries. *JAMA* 2005;294:2611-7.
54. Davis DH, Localio AR, Stafford PW, Helfaer MA, Durbin DR. Trends in operative management of pediatric splenic injury in a regional trauma system. *Pediatrics* 2005;115:89-94.
55. Demetriades D, Martin M, Salim A, Rhee P, Brown C, Chan L, et al. The effect of trauma center designation and trauma volume on outcome in specific severe injuries. *Annals of Surgery* 2005;242:512-9.
56. Holmes JHt, Wiebe DJ, Tataria M, Mattix KD, Mooney DP, Scaife ER, et al. The failure of nonoperative management in pediatric solid organ injury: a multi-institutional experience. *J Trauma* 2005;59:1309-13.
57. Winthrop AL, Brasel KJ, Stahovic L, Paulson J, Schneeberger B, Kuhn EM. Quality of life and functional outcome after pediatric trauma. *J Trauma* 2005;58:468-73; discussion 73-4.
58. Demetriades D, Martin M, Salim A, Rhee P, Brown C, Doucet J, et al. Relationship between American College of Surgeons trauma center designation and mortality in patients with severe trauma (injury severity score > 15). *J Am Coll Surg* 2006;202:212-5; quiz A45.
59. Densmore JC, Lim HJ, Oldham KT, Guice KS. Outcomes and delivery of care in pediatric injury. *J Pediatr Surg* 2006;41:92-8; discussion -8.
60. Grinkeviciūte D, Kevalas R, Tamasauskas A, Matukevicius A, Gurskis V, Liesiene R. [Severe pediatric head injury: is there any optimal solution?]. *Medicina (Kaunas)* 2006;42:278-87.
61. Javouhey E, Guérin AC. [Organisation of health care management for children with traumatic brain or spinal injuries]. *Arch Pediatr* 2006;13:557-9.
62. Junkins EP, O'Connell KJ, Mann NC. Pediatric Trauma Systems in the United States: Do They Make a Difference? *Clinical Pediatric Emergency Medicine* 2006;7:76-81.
63. Mitra B, Cameron PA, Butt W, Rosenfeld JV. Children or young adults? A population-based study on adolescent head injury. *Anz Journal of Surgery* 2006;76:343-50.
64. Mooney DP, Rothstein DH, Forbes PW. Variation in the management of pediatric splenic injuries in the United States. *J Trauma* 2006;61:330-3; discussion 3.
65. Mullins RJ, Trunkey DD. Variation in treatment of pediatric spleen injury at trauma centers versus nontrauma centers. *J Am Coll Surg* 2006;203:263; author reply 4.
66. Stylianos S, Egorova N, Guice KS, Arons RR, Oldham KT. Variation in treatment of pediatric spleen injury at trauma centers versus nontrauma centers: a call for dissemination of American Pediatric Surgical Association benchmarks and guidelines. *J Am Coll Surg* 2006;202:247-51.
67. Culica D, Aday LA, Rohrer JE. Regionalized trauma care system in Texas: implications for redesigning trauma systems. *Med Sci Monit* 2007;13:Sr9-18.
68. Groner JI, Covert J, Lowell WL, Hayes JR, Nwomeh BC, Caniano DA. The impact of managing moderately injured pediatric trauma patients without immediate surgeon presence. *J Pediatr Surg* 2007;42:1026-9; discussion 9-30.
69. Klinkner DB, Arca MJ, Lewis BD, Oldham KT, Sato TT. Pediatric vascular injuries: patterns of injury, morbidity, and mortality. *Journal of Pediatric Surgery* 2007;42:178-83.
70. Newgard CD, McConnell KJ, Hedges JR, Mullins RJ. The benefit of higher level of care transfer of injured patients from nontertiary hospital emergency departments. *Journal of Trauma* 2007;63:965-71.
71. Ochoa C, Chokshi N, Upperman JS, Jurkovich GJ, Ford HR. Prior studies comparing outcomes from trauma care at children's hospitals versus adult hospitals. *Journal of Trauma-Injury Infection and Critical Care* 2007;63:S87-S91.

© 2023 Moore L et al. *JAMA Network Open.*

72. Wang NE, Chan J, Mahlow P, Wise PH. Trauma center utilization for children in California 1998-2004: trends and areas for further analysis. *Acad Emerg Med* 2007;14:309-15.
73. DuBose JJ, Browder T, Inaba K, Teixeira PG, Chan LS, Demetriades D. Effect of trauma center designation on outcome in patients with severe traumatic brain injury. *Arch Surg* 2008;143:1213-7; discussion 7.
74. Hartman M, Watson RS, Linde-Zwirble W, Clermont G, Lave J, Weissfeld L, et al. Pediatric traumatic brain injury is inconsistently regionalized in the United States. *Pediatrics* 2008;122:E172-E80.
75. Pracht EE, Tepas JJ, Langland-Orban B, Simpson L, Pieper P, Flint LM. Do pediatric patients with trauma in Florida have reduced mortality rates when treated in designated trauma centers? *Journal of Pediatric Surgery* 2008;43:212-21.
76. Scarborough K, Slone DS, Uribe P, Craun M, Bar-Or R, Bar-Or D. Reduced mortality at a community hospital trauma center: the impact of changing trauma level designation From II to I. *Arch Surg* 2008;143:22-7; discussion 7-8.
77. Squyer E, Cherry RA, Lehman E, Yanturali S, Kilicaslan I, Oktay C, et al. Comparison of trauma mortality between two hospitals in Turkey to one trauma center in the US. *European Journal of Emergency Medicine* 2008;15:209-13.
78. Cooper Z, Rivara FP, Wang J, MacKenzie EJ, Jurkovich GJ. Withdrawal of life-sustaining therapy in injured patients: variations between trauma centers and nontrauma centers. *J Trauma* 2009;66:1327-35.
79. Foley DS, Draus JM, Jr., Santos AP, Franklin GA. An analysis of risk-taking behavior among adolescent blunt trauma patients. *J Ky Med Assoc* 2009;107:170-5.
80. Kejriwal R, Civil I. Time to definitive care for patients with moderate and severe traumatic brain injury--does a trauma system matter? *N Z Med J* 2009;122:40-6.
81. Nance ML, Carr BG, Branas CC. Access to Pediatric Trauma Care in the United States. *Archives of Pediatrics & Adolescent Medicine* 2009;163:512-8.
82. Petrosyan M, Guner YS, Emami CN, Ford HR. Disparities in the delivery of pediatric trauma care. *Journal of Trauma* 2009;67:S114-9.
83. Prentiss KA, Vinci R. Children in emergency departments: who should provide their care? *Archives of Disease in Childhood* 2009;94:573-6.
84. Siram S, Oyetunji TA, Khoury AL, Walker SR, Bolorunduro OB, Chang DC, et al. Pediatric Trauma at an Adult Trauma Center. *Journal of the National Medical Association* 2010;102:692-5.
85. Odetola F, Gebremariam A. Pediatric trauma care in the U.S: Patterns of emergency department visits, and variation in hospital outcomes and resource use by hospital trauma center status. *Critical Care Medicine* 2011;39:159.
86. Deasy C, Gabbe B, Palmer C, Babl FE, Bevan C, Crameri J, et al. Paediatric and adolescent trauma care within an integrated trauma system. *Injury* 2012;43:2006-11.
87. Hsiao M, Haas B, Gomez D, De Mestral C, Sharma S, Langer JC, et al. Population-based analysis of blunt splenic injuries in children: Using operative rate as a quality of care indicator. *Journal of Surgical Research* 2012;172:254-5.
88. Janssens L, Holtslag HR, van Beeck EF, Leenen LP. The effects of regionalization of pediatric trauma care in the Netherlands: a surveillance-based before-after study. *J Trauma Acute Care Surg* 2012;73:1284-7.
89. Notrica DM, Weiss J, Garcia-Filion P, Kuroiwa E, Clarke D, Harte M, et al. Pediatric trauma centers: Correlation of ACS-verified trauma centers with CDC statewide pediatric mortality rates. *Journal of Trauma & Acute Care Surgery* 2012;73:566-72.
90. Hannon E, Potter S, Jaiganesh T, Muhktar Z, Okoye B. The impact of adult major trauma centre status on paediatric trauma activity. *Emergency Medicine Journal* 2013;30:828-30.
91. Hartin CW, Jordan JM, Gemme S, Glick PL, Caty MG, Ozgediz DE, et al. Computed tomography scanning in pediatric trauma: Opportunities for performance improvement and radiation safety. *Journal of Surgical Research* 2013;180:226-31.
92. Kernic MA, Rivara FP, Zatzick DF, Bell MJ, Wainwright MS, Groner JI, et al. Triage of Children with Moderate and Severe Traumatic Brain Injury to Trauma Centers. *Journal of Neurotrauma* 2013;30:1129-36.
93. Kharbanda AB, Flood A, Blumberg K, Kreykes NS. Analysis of radiation exposure among pediatric trauma patients at national trauma centers. *Journal of Trauma & Acute Care Surgery* 2013;74:907-11.

© 2023 Moore L et al. *JAMA Network Open.*

94. Marin JR, Weaver MD, Barnato AE, Yealy DM, Roberts MS. Emergency department variation in head computed tomography scanning among pediatric patients with head trauma. *Academic Emergency Medicine* 2013;20:S234-S5.
95. Mooney DP, Gutierrez IM, Chen Q, Forbes PW, Zurakowski D. Impact of trauma system development on pediatric injury care. *Pediatr Surg Int* 2013;29:263-8.
96. Van Cleve W, Kernic MA, Ellenbogen RG, Wang J, Zatzick DF, Bell MJ, et al. National Variability in Intracranial Pressure Monitoring and Craniotomy for Children With Moderate to Severe Traumatic Brain Injury. *Neurosurgery* 2013;73:746-52.
97. Adelgais KM, Browne L, Holsti M, Metzger RR, Murphy SC, Dudley N. Cervical spine computed tomography utilization in pediatric trauma patients. *Journal of Pediatric Surgery* 2014;49:333-7.
98. Agarwal S, Jokerst C, Siegel M, Hildebolt C. Monitoring radiation exposures from pediatric CT in child visits to pediatric and adult-focused emergency departments. *Pediatric Radiology* 2014;44:S91-S2.
99. Anders JF, Adelgais K, Hoyle JD, Jr., Olsen C, Jaffe DM, Leonard JC. Comparison of outcomes for children with cervical spine injury based on destination hospital from scene of injury. *Acad Emerg Med* 2014;21:55-64.
100. Gaines BA. Teen trauma without the drama: Outcomes of adolescents treated at Ohio adult versus pediatric trauma centers EDITORIAL CRITIQUE. *Journal of Trauma and Acute Care Surgery* 2014;77:116-.
101. Hsiao M, Sathya C, de Mestral C, Langer JC, Gomez D, Nathens AB. Population-based analysis of blunt splenic injury management in children: operative rate is an informative quality of care indicator. *Injury* 2014;45:859-63.
102. Iqbal CW, St Peter SD, Tsao K, Cullinane DC, Gourlay DM, Ponsky TA, et al. Operative vs Nonoperative Management for Blunt Pancreatic Transection in Children: Multi-Institutional Outcomes. *Journal of the American College of Surgeons* 2014;218:157-62.
103. Johnson KN, Harte M, Garcia-Filion P, Notrica DM. Fate of the combined adult and pediatric trauma centers: impact of increased pediatric trauma requirements. *American Surgeon* 2014;80:1280-2.
104. Paull B. Teen trauma without the drama: Outcomes of adolescents treated at ohio adult versus pediatric trauma centers: Walther AE, Pritts TA, Falcone RA, et al. J Trauma Acute Care Surg 2014;77:109-16; Discussion 116. *Journal of Emergency Medicine* 2014;47:502.
105. Rialon K, Englum BR, Gulack BC, Bhattacharya SD, Talbot L, Shapiro ML, et al. Utilization of computed tomography in pediatric trauma: A comparison of trauma centers using the National Trauma Data Bank. *Journal of the American College of Surgeons* 2014;219:e130.
106. Sarkar K, Keachie K, Nguyen U, Muizelaar JP, Zwienenberg-Lee M, Shahlaie K. Computed tomography characteristics in pediatric versus adult traumatic brain injury. *Journal of Neurosurgery-Pediatrics* 2014;13:307-14.
107. Sharp NE, Svetanoff WJ, Desai A, Alemayehu H, Raghavan MU, Sharp SW, et al. Radiation exposure from head computed tomography scans in pediatric trauma. *J Surg Res* 2014;192:276-9.
108. Alarhayem AQ, Liao LF, Stewart RM, Myers JG, Eastridge BJ, Nicholson SE, et al. Management of pediatric splenic injuries: A nationwide analysis. *Journal of the American College of Surgeons* 2015;221:e143-e4.
109. Ashley DW, Pracht EE, Medeiros RS, Atkins EV, Nesmith EG, Johns TJ, et al. An analysis of the effectiveness of a state trauma system: Treatment at designated trauma centers is associated with an increased probability of survival. *Journal of Trauma and Acute Care Surgery* 2015;78:706-14.
110. Capecci LM, Jeremitsky E, Smith RS, Philp F. Trauma centers with higher rates of angiography have a lesser incidence of splenectomy in the management of blunt splenic injury. *Surgery* 2015;158:1020-4; discussion 4-6.
111. Farach SM, Danielson PD, Amankwah EK, Chandler NM. Admission patterns in pediatric trauma patients with isolated injuries. *J Surg Res* 2015;198:13-8.
112. Kelly ML, Banerjee A, Nowak M, Steinmetz M, Claridge JA. Decreased mortality in traumatic brain injury following regionalization across hospital systems. *J Trauma Acute Care Surg* 2015;78:715-20.
113. Khalil M, Rhee PM, Haider AA, Kulvatunyou N, Zangbar B, O'Keeffe T, et al. Take 'em where they belong: Outcome differences in pediatric traumatic brain injury among trauma centers. *Journal of the American College of Surgeons* 2015;221:S108-S9.
114. Liu S, Bowman SM, Smith TC, Sharar SR. Trends in pediatric spleen management: Do hospital type and ownership still matter? *J Trauma Acute Care Surg* 2015;78:935-42.

115. Skinner DL, den Hollander D, Laing GL, Rodseth RN, Muckart DJ. Severe blunt thoracic trauma: differences between adults and children in a level I trauma centre. *S Afr Med J* 2015;105:47-51.
116. Stiles PJ, Helmer SD, Ward JG, Reyes J, Harrison PB, Haan JM. Pediatric trauma system models: do systems using adult trauma surgeons exclusively compare favorably with those using pediatric surgeons after initial resuscitation with an adult trauma surgeon? *American Journal of Surgery* 2015;210:1063-8.
117. Webman RB, Carter EA, Mittal S, Wang J, Madigan D, Burd RS. Evaluating the association of center designation and in-hospital mortality among injured adolescents. *Journal of the American College of Surgeons* 2015;221:e141.
118. Amram O, Schuurman N, Pike I, Friger M, Yanchar NL. Assessing access to paediatric trauma centres in Canada, and the impact of the golden hour on length of stay at the hospital: an observational study. *BMJ Open* 2016;6:e010274.
119. Baudin F, Floccard B, Desgranges FP, Courtil-Teyssedre S, De Queiroz M, Richard N, et al. Effectiveness of management of children with severe trauma in a pediatric trauma center and in an adult trauma center: A before-after study. *Archives De Pediatrie* 2016;23:367-72.
120. Falcone RA, Milliken WJ, Bensard DD, Haas L, Daugherty M, Gray L, et al. A paradigm for achieving successful pediatric trauma verification in the absence of pediatric surgical specialists while ensuring quality of care. *Journal of Trauma & Acute Care Surgery* 2016;80:433-9.
121. Pandit V, Hassan A, Azim A, Rhee PM, O'Keeffe T, Kulvatunyou N, et al. Pediatric Traumatic Brain Injury in Adult vs Pediatric Trauma Centers: What are We Doing Different? *Journal of the American College of Surgeons* 2016;223:E202-E3.
122. Pandit V, Ibraheem K, Rhee PM, Jokar TO, O'Keeffe T, Vercruysse GA, et al. Assessing Variability Among Pediatric and Adult Trauma Centers in Managing Colonic Injury. *Journal of the American College of Surgeons* 2016;223:E196-E.
123. Puckett Y, Bonacorsi L, Caley M, Farmakis S, Fitzpatrick C, Chatoorgoon K, et al. Imaging before transfer to designated pediatric trauma centers exposes children to excess radiation. *Journal of Trauma & Acute Care Surgery* 2016;81:229-35.
124. Khan M, Jehan F, O'Keeffe T, Pandit V, Kulvatunyou N, Tang A, et al. Primary repair for pediatric colonic injury: Are there differences among adult and pediatric trauma centers? *Journal of Surgical Research* 2017;220:176-81.
125. Snyder CW, Chandler NM, Litz CN, Pracht EE, Danielson PD, Ciesla DJ. Immature patients in a mature system: Regional analysis of Florida's pediatric trauma system. *Journal of Trauma and Acute Care Surgery* 2017;83:711-5.
126. Bone MC, Schlosser MJ, Perlin JB. Re: Injured Children Receive Twice the Radiation Dose at Nonpediatric Trauma Centers Compared With Pediatric Trauma Centers. *Journal of the American College of Radiology* 2018;15:825-.
127. Elkbuli A, Lopez JR, Perales Villarroel P, Ang D, Huazhi LIU, Boneva D, et al. Comparison of Outcomes for Pediatric Trauma at Different Types of Trauma Centers: The Unresolved Mystery. *American Surgeon* 2018;84:1054-7.
128. Filipescu R, Powers C, Rothstein DH, Harmon CM, Bass KD. Pediatric vs Adult Trauma Centers: Closing the Gap in Nonoperative Management of Splenic Injury: A National Trauma Data Bank Study. *Journal of the American College of Surgeons* 2018;227:S270-S.
129. Gorelik M, Elkbuli A, Hai S, Torres A, McKenney M. Atraumatic Transition from a Pediatric Emergency Center to a Pediatric Trauma Center: A Fight for Better Outcomes. *American Surgeon* 2018;84:1489-92.
130. Hadjipavlou M, Grouse E, Gray R, Sri D, Huang D, Brown C, et al. Managing penetrating renal trauma: experience from two major trauma centres in the UK. *BJU Int* 2018;121:928-34.
131. He JC, Sajankila N, Kreiner LA, Allen DL, Claridge JA. Level I Trauma Centers: More Is Not Necessarily Better. *Am Surg* 2018;84:557-64.
132. Herrera-Escobar JP, Rios-Diaz AJ, Zogg CK, Wolf LL, Harlow A, Schneider EB, et al. The "mortality ascent": Hourly risk of death for hemodynamically unstable trauma patients at Level II versus Level I trauma centers. *J Trauma Acute Care Surg* 2018;84:139-45.
133. Horst MA, Jammula S, Gross BW, Cook AD, Bradburn EH, Altenburg J, et al. Undertriage in trauma: Does an organized trauma network capture the major trauma victim? A statewide analysis. *J Trauma Acute Care Surg* 2018;84:497-504.

© 2023 Moore L et al. *JAMA Network Open.*

134. Lodwick DL, Cooper JN, Gonzalez DO, Lawrence AE, Lee C, Krishnamurthy R, et al. Disparities in Radiation Burden from Trauma Evaluation at Pediatric Versus Nonpediatric Institutions. *Journal of Surgical Research* 2018;232:475-83.
135. Mackel CE, Morel BC, Winer JL, Park HG, Sweeney M, Heller RS, et al. Secondary overtriage of pediatric neurosurgical trauma at a Level I pediatric trauma center. *Journal of Neurosurgery-Pediatrics* 2018;22:375-83.
136. Nabaweesi R, Ramakrishnaiah RH, Aitken ME, Rettiganti MR, Luo CQ, Maxson RT, et al. Injured Children Receive Twice the Radiation Dose at Nonpediatric Trauma Centers Compared With Pediatric Trauma Centers. *Journal of the American College of Radiology* 2018;15:58-64.
137. Notrica DM, Sayrs LW, Krishna N. The effect of verified pediatric trauma centers, state laws, and crash characteristics on time trends in adolescent motor vehicle fatalities, 1999-2015. *Journal of Trauma and Acute Care Surgery* 2018;85:944-52.
138. Schlegel C, Greeno A, Chen H, Raees MA, Collins KF, Chung DH, et al. Evolution of a level I pediatric trauma center: Changes in injury mechanisms and improved outcomes. *Surgery* 2018;163:1173-7.
139. Strohm PC, Zwingmann J, Bayer J, Neumann MV, Lefering R, Schmal H, et al. Differences in the outcome of seriously injured children depending on treatment level. *Unfallchirurg* 2018;121:306-12.
140. Talbot C, Davis N, Majid I, Young M, Bouamra O, Lecky FE, et al. Fractures of the femoral shaft in children: national epidemiology and treatment trends in England following activation of major trauma networks. *Bone Joint J* 2018;100-b:109-18.
141. Tessler RA, Lyons VH, Hagedorn JC, Vavilala MS, Goldin A, Arbabi S, et al. Transfer and nontransfer patients in isolated low-grade blunt pediatric solid organ injury: Implications for regionalized trauma systems. *J Trauma Acute Care Surg* 2018;84:606-12.
142. Dakessian A, Bachir R, El Sayed M. Impact of trauma designation levels on survival of drowning victims: An observational study from trauma centers in the United States. *Medicine (Baltimore)* 2019;98:e17721.
143. Dixon MD, Engum S. Pediatric Trauma System Evaluation before and after Level II Verification. *American Surgeon* 2019;85:1281-7.
144. Ma LW, Hatchimonji JS, Kaufman EJ, Sharoky CE, Smith BP, Holena DN. Failure to rescue as a center-level metric in pediatric trauma. *Surgery* 2019;165:1116-21.
145. Sathya C, Alali AS, Wales PW, Langer JC, Kenney BD, Burd RS, et al. Computed tomography rates and estimated radiation-associated cancer risk among injured children treated at different trauma center types. *Injury-Int J Care Inj* 2019;50:142-8.
146. Tang OY, Yoon JS, Kimata AR, Lawton MT. Volume-outcome relationship in pediatric neurotrauma care: analysis of two national databases. *Neurosurg Focus* 2019;47:11.
147. Vassallo J, Webster M, Barnard EBG, Lyttle MD, Smith JE. Epidemiology and aetiology of paediatric traumatic cardiac arrest in England and Wales. *Arch Dis Child* 2019;104:437-43.
148. Vogel AM, Zhang JW, Mauldin PD, Williams RF, Huang EY, Santore MT, et al. Variability in the evalution of pediatric blunt abdominal trauma. *Pediatric Surgery International* 2019;35:479-85.
149. Green PA, Wilkinson DJ, Bouamra O, Fragoso M, Farrelly PJ. Variations in the management of adolescents with blunt splenic trauma in England and Wales: are we preserving enough? *Annals of the Royal College of Surgeons of England* 2020;102:488-92.
150. Hawkins RB, Raymond SL, Hamann HC, Taylor JA, Mustafa MM, Islam S, et al. Outcomes After Prehospital Endotracheal Intubation in Suburban/Rural Pediatric Trauma. *Journal of Surgical Research* 2020;249:138-44.
151. Mahran A, Fernstrum A, Swindle M, Mishra K, Bukavina L, Raina R, et al. Impact of trauma center designation in pediatric renal trauma: National Trauma Data Bank analysis. *J Pediatr Urol* 2020;16:658.e1-.e9.
152. Prieto JM, Van Gent JM, Calvo RY, Checchi KD, Wessels LE, Sise CB, et al. Evaluating surgical outcomes in pediatric extremity vascular trauma. *Journal of Pediatric Surgery* 2020;55:319-23.
153. Sheehan BM, Grigorian A, Maithel S, Borazjani B, Fujitani RM, Kabutey NK, et al. Penetrating Abdominal Aortic Injury: Comparison of ACS-Verified Level-I and II Trauma Centers. *Vasc Endovascular Surg* 2020;54:692-6.
154. Strait L, Sussman R, Ata A, Edwards MJ. Utilization of CT imaging in minor pediatric head, thoracic, and abdominal trauma in the United States. *Journal of Pediatric Surgery* 2020;55:1766-72.

© 2023 Moore L et al. *JAMA Network Open.*

155. Cutler GJ, Zagel AL, Spaulding AB, Linabery AM, Kharbanda AB. Emergency Department Visits for Pediatric Firearm Injuries by Trauma Center Type. *Pediatr Emerg Care* 2021;37:e686-e91.
156. Evans CCD, Li W, Seitz D. Injury-related deaths in the Ontario provincial trauma system: a retrospective population-based cohort analysis. *CMAJ open* 2021;9:E208-E14.
157. Hrdy M, Mahesh M, Miller M, Klein B, Stewart D, Ryan LM. An Analysis of Computed Tomography-Related Radiation Exposure in Pediatric Trauma Patients. *Pediatric Emergency Care* 2021;37:296-302.
158. Kim W, Ahn N, Ata A, Adamo MA, Entezami P, Edwards M. Pediatric cervical spine injury in the United States: Defining the burden of injury, need for operative intervention, and disparities in imaging across trauma centers. *Journal of Pediatric Surgery* 2021;56:293-6.
159. Lewis SL, Grimes AD, Stewart KE, Landman A, Sarwar Z, Johnson JJ, et al. Retrospective review of intervention for traumatic blunt spleen injuries in adolescents by trauma center type. *Am J Surg* 2021.
160. Haratian A, Grigorian A, Rajalingam K, Dolich M, Schubl S, Kuza CM, et al. Laparoscopy in the Evaluation of Blunt Abdominal Injury in Level-I and II Pediatric Trauma Centers. *Am Surg* 2022:31348211033535.
161. Keane OA, Escobar MA, Neff LP, Mitchell IC, Chern JJ, Santore MT. Pediatric Mild Traumatic Brain Injury: Who Can Be Managed at a Non-pediatric Trauma Center Hospital? A Systematic Review of the Literature. *American Surgeon* 2022;88:447-54.
162. Yung KK, Ardern CL, Serpiello FR, Robertson S. Characteristics of Complex Systems in Sports Injury Rehabilitation: Examples and Implications for Practice. *Sports Med Open* 2022;8:24.

© 2023 Moore L et al. *JAMA Network Open.*

eTable 5. Characteristics of Included Studies

First author Year	Country Study period	Setting	Population	Intervention Type of center; designation level; verification status	Comparator Type of center; designation level; verification status	Outcomes
Gerber 2023[1]	USA 2011-2015	ACS-verified level I-II trauma centers submitting to the NTDB	Children **< 18 yoa** with isolated mild traumatic brain injury	PTC; I-II; ACS	ATC; I-II; ACS	Head CT*
Derderian 2022[2]	USA 2016-2018	Trauma centers participating in TQIP	Adolescents **16 - 19 yoa** with **blunt solid organ injuries**	PTC; nr; nr	ATC; nr; nr	Mortality*<br>ICU admission<br>Hospital LOS<br>Operative management<br>RBC transfusions
Hairr 2022[3]	USA 2017-2017	Trauma centers participating in TQIP	Children < **19 yoa** admitted following **injury**	PTC with <600 beds	Non-designated with <600 beds	Mortality*
Lewit 2022[4]	USA 2015	Trauma centers submitting to the NTDB	Children **< 17 yoa** with **isolated head injuries**	PTC; nr; nr	ATC; nr; nr<br>CTC; nr; nr	Mortality*<br>ICU admission<br>LOS<br>Ventilator use
Killien 2022[5]	USA 2007-2016	Level I and II trauma centers submitting to the NTDB	Children **<18 yoa** with **isolated severe traumatic brain injury**	PTC; I-II; state ± ACS	ATC; I-II; state ± ACS	Tracheostomy*
Pulido 2022[6]	USA 2003-2018	All ACS-verified level I-II trauma centers in Pennsylvania (PTOS)	Children < **15 yoa** with **liver injuries**	PTC; I-II; ACS	ATC; I-II; ACS	Mortality*<br>Hospital LOS<br>ICU LOS<br>Operative management*<br>Functional status on discharge
Sheff 2022[7]	USA 2013-2017	Trauma centers participating in TQIP	Adolescents **15 - 17 yoa** with **isolated severe traumatic brain injury**	PTC; I; state ± ACS	ATC; I; state ± ACS<br>CTC; I; state ± ACS	Mortality*<br>ICU admission*
Stephenson 2022[8]	USA 2016-2019	ACS-verified level I-II trauma centers submitting to the NTDB	Adolescents **14 - 18 yoa** admitted following injury	PTC; I-II; ACS	ATC; I-II; ACS<br>CTC; I-II; ACS	Mortality*
Ali 2021[9]	USA 2013-2015	Level I and II trauma centers submitting to the NTDB	Children **≤ 14yoa** with **pelvic fractures**	PTC; I-II; state ± ACS	ATC; I-II; state ± ACS	Mortality*<br>Complications*<br>Abdomen/pelvis CT*<br>Head CT*

© 2023 Moore L et al. *JAMA Network Open.*

						Thoracic CT*<br>Whole body CT*
Evans<br>2021[10]	UK<br>2012-2018	All major trauma centers in the UK (TARN)	Adolescents **14 - 18 yoa** with **moderate to severe injuries** (TARN inclusion criteria)	PTC; major trauma centers; UK MoH	ATC; major trauma centers; UK MoH<br>CTC; major trauma centers; UK MoH	Mortality*
Khalil<br>2021[11]	USA<br>2011-2012	Level I-IV trauma centers submitting to the NTDB	Children **< 19 yoa** with **severe injuries** (ISS>15)	PTC; I-II; state ± ACS	ATC; I-IV; state ± ACS	ED mortality*<br>IP mortality*<br>IP complications*<br>Hospital LOS<br>ICU-free LOS<br>Ventilator days<br>Discharge disposition
Kim, 2021[12]	USA<br>2016	Trauma centers submitting to the NTDB	Children **< 15 yoa** with **cervical spine injury**	PTC	ATC; I-III; state ± ACS<br>CTC; I-III; state ± ACS<br>Non-designated	Operative management<br>Imaging
Massoumi<br>2021[13]	USA<br>2015-2016	Trauma centers participating in TQIP	Children **< 18 yoa** admitted following **injury**	PTC; I; state ± ACS<br>PTC; all; state ± ACS	ATC; I; state ± ACS<br>ATC; I-IV; state ± ACS<br>Non -designated	Cervical spine CT*<br>Cervical spine MRI<br>Cervical spine X-ray
Scantling<br>2021[14]	USA<br>2007-2017	Stand-alone urban PTC and ATC in Pittsburgh and Philadelphia (PTOS)	Children **14-18 yoa** with **firearm injuries**	PTC; I; nr	ATC; I-II; nr	Mortality*
Swendiman<br>2021a[15]	USA<br>2010-2016	ACS-verified level I-II trauma centers submitting to the NTDB	Children **13-16 yoa** with **firearm injuries**	PTC; I-II; ACS	ATC; I-II; ACS<br>CTC; I-II; ACS	Mortality*
Swendiman<br>2021b[16]	USA<br>2010-2015	ACS-verified level I&II trauma centers submitting to the NTDB	Children **< 18 yoa** with **blunt splenic injuries**	PTC; I-II; ACS	ATC; I-II; ACS<br>CTC; I-II; ACS	Angioembolization*<br>Splenectomy*
Yanchar<br>2021[17]	Canada<br>2012-2016	One PTC and one ATC in southern Alberta trauma system	Adolescents **15-17 yoa** with **intra-abdominal injury, femur fracture or TBI**	PTC; I; provincial	ATC; I; provincial	Whole body CT
Filipescu<br>2020[18]	USA<br>2011-2015	ACS-verified level I-II trauma centers submitting to the NTDB	Children **0-18 yoa** with **splenic injuries**	PTC; I-II; state ± ACS	ATC; I-II; state ± ACS<br>CTC; I-II; state ± ACS	Operative management*

© 2023 Moore L et al. *JAMA Network Open.*

Hatchimonji 2020[19]	USA 2010-2016	ACS-verified level I trauma centers submitting to the NTDB	Children **< 20 yoa** with **firearm injuries**	PTC; I; ACS	ATC; I; ACS<br>CTC; I; ACS	Mortality*
Prieto 2020[20]	USA 2016	ACS-verified level I-II trauma centers submitting to the NTDB	Children **< 17 yoa** with **extremity vascular injuries**	PTC; I-II; ACS	CTC; I-II; ACS	Surgical amputation*
Prieto, 2020[21]	USA 2007-2014	All trauma centers in California	Children **< 18 yoa** with **extremity arterial injury**	PTC	ATC	Mortality<br>Amputation
Strait, 2020[22]	USA 2014-2016	Trauma centers submitting to the NTDB	Children **< 15 yoa** with **minor injury**	ACS; I-II; PTC	ACS; I-III; state ± ACS	CT scans
Swendiman 2020[23]	USA 2010-2014	ACS-verified level I-II trauma centers submitting to the NTDB	Children **< 10 yoa** with **isolated blunt solid organ injuries**	PTC; I-II; ACS	ATC; I-II; ACS<br>CTC; I-II; ACS	Interventional radiology*
Yung 2020[24]	USA 2007-2016	ACS-verified level I and II trauma centers submitting to the NTDB	Children **< 20 yoa** with **isolated liver and spleen injuries**	PTC; I-II; ACS	ATC; I-II; ACS<br>CTC; I-II; ACS	Operative management*
Alexander 2019[25]	USA 2004-2017	ACS-verified level I trauma center in Iowa	Children **< 18 yoa** admitted for a **blunt splenic injuries**	PTC; I; ACS	ATC; I; ACS	Average hospital charges<br>Average professional charges
Myers 2019[26]	USA 2006	All US hospitals (HCUP)	Children **< 17 yoa** with **injury diagnostic code**	PTC; I-II; state ± ACS	ATC; I-II; state ± ACS	Mortality*
Sathya, 2019[27]	USA 2010-2013	Trauma centers participating in TQIP	Children **< 19 yoa** with **blunt moderate to major trauma**	PTC	CTC<br>ATC	CT imaging
Vassallo, 2019[28]	GB	All major trauma centers and trauma units in GB (TARN)	Children **< 19 yoa** with traumatic cardiac arrest	PTC or CTC	ATC	Mortality
Bardes 2018[29]	USA 2007-2014	ACS-verified level I trauma centers submitting to the NTDB	Children **< 15 yoa** with **isolated, blunt, severe TBI**	PTC; I; ACS	ATC; I; ACS<br>CTC; I; ACS	Mortality*
Schlegel, 2018[30]	USA 2005-2016	ATC that became ACS-verified PTC	Children **< 16 yoa** presenting as level I trauma activations	PTC	ATC	Mortality<br>ICU admission<br>Operative management
Adams 2017[31]	Australia 2000-2011	All hospital in New South Wales	Children **0–16 yoa** with **blunt splenic injuries**	PTC; NSW MoH	ATC; metropolitan or rural; NSW MoH<br>Local health facility; metropolitan or rural; NSW MoH	Splenectomy*

© 2023 Moore L et al. *JAMA Network Open.*

Mitchell 2017[32]	Australia 2009-2014	Trauma centers submitting to the New South Wales Trauma Registry	Trauma patient **< 16 yoa** hospitalised with **major trauma** (ISS > 12)	PTC; I; MoH	ATC; I; MoH<br>ATC; II-III; MoH	Mortality*
Miyata 2017a[33]	USA 2007-2014	Level I or II centers submitting to the NTDB	Children **<19 yoa** with grade III-IV **blunt splenic injuries**	PTC; I-II; state ± ACS	ATC; I-II; state ± ACS<br>CTC; I-II; state ± ACS	Mortality*<br>Splenic surgery*<br>Total splenectomy*
Miyata 2017b[34]	USA 2007-2012	Trauma centers submitting to the NTDB	Children **≤ 18 yoa** with **penetrating injuries**	PTC; nr; state ± ACS	ATC; nr; state ± ACS	Mortality*<br>Discharge destination
Baudin, 2016[35]	France 2005-2007	Un CTC and a PTC in Lyon	Children **1-15 yoa** admitted for **major trauma**	PTC	CTC	Early mortality<br>Time to CT
Pandit 2016[36]	USA 2011-2012	Level I-II trauma centers submitting to the NTDB	Patients **< 18 yoa**, with **head, chest, or abdominal & pelvic CT**	PTC; I-II; nr	ATC; I-II; nr	Whole body CT*
Safavi 2016[37]	USA 2011-2012	ACS-verified level I-II ATC and PTC submitting to the NTDB	Patients **<18 yoa** with **isolated blunt solid organ injuries**	PTC; I-II; ACS	ATC; I-II; ACS	Splenic surgery*
Walther 2016[38]	USA 2007-2011	ACS-verified level I ATC and PTC submitting to the NTDB	Adolescents **15–19 yoa** with **LOS> 1 day**	PTC; I; ACS	ATC; I; ACS	Mortality*
Webman 2016[39]	USA 2010	Level I-II trauma centers submitting to the NTDB	Patients **15-19 yoa** with **injuries**	PTC; I-II; nr	ATC; I-II; nr<br>CTC; I-II; nr	Mortality*<br>In-hospital mortality<br>Discharge to rehabilitation
Kelley-Quon 2015[40]	USA 2000-2013	Community hospital that became level II PTC in Los Angeles	Children **< 15 yoa** admitted for **injury**	PTC; II;	ATC; II; nr	CT imaging*
Marin 2015[41]	USA 2010-2013	14 network-affiliated EDs in Pittsburgh	Children **< 19 yoa** with an **injury diagnosis**	PTC; I; nr	Non-designated	Any CT*<br>Abdominal CT*<br>Head CT*<br>C-spine CT*<br>Chest CT*
Sathya 2015[42]	USA 2010-2013	Level I-II trauma centers participating in TQIP	Children **< 19 yoa** with **moderate-severe injuries** (AIS ≥ 2)	PTC;I-II; ACS or state	ATC;I-II; ACS or state<br>CTC;I-II; ACS or state	Mortality*
Walther 2014[43]	USA 2008-2012	Level I-II trauma centers in Ohio	Children **15-19 yoa** with **LOS≥1 day**	PTC;I-II; ACS	ATC; I; ACS	Mortality*<br>Hospital LOS<br>ICU LOS<br>Ventilator days
Lippert 2013[44]	USA 1999-2010	Level I ATC and PTC in New York State	Adolescents **14-17 yoa** with **blunt splenic injuries**	PTC; I; nr	ATC; I; nr	Splenectomy*

© 2023 Moore L et al. *JAMA Network Open.*

Matsushima 2013a[45]	USA 2005-2010	Trauma centers in Pennsylvania (PTOS)	Adolescents **13-18 yoa** with **moderate to severe injuries** (LOS>48hrs, transfer-in, ICU unit admission, or death)	PTC; **I**; state ± ACS	ATC; **I-III**; state ± ACS	Mortality*<br>Complications*<br>CT<br>Laparotomy*
Matsushima 2013b[46]	USA 2005-2010	Trauma centers in Pennsylvania (PTOS)	Adolescents **13-18 yoa** with **blunt spleen, liver, or kidney injuries**	PTC; nr; state ± ACS	ATC; nr; state ± ACS	Splenic surgery*
Mitchell 2013[47]	Australia 2003-2008	Trauma centers in New South Wales	Children **< 16 yoa** with **severe injuries** (ISS > 15)	PTC; **I**; MoH	ATC; **I**; MoH<br>ATC; **II-III** or non-designated; MoH	Mortality*
Wang 2013[48]	USA 1999-2014	All hospitals in California (patient discharge data)	Children **< 19 yoa** with **injury diagnostic code**	PTC; **I-II**; state ± ACS	ATC & CTC; **I-II**; state ± ACS	Mortality*
Amini 2011[49]	Canada 1998-2005	58 designated trauma centers in regionalized trauma system in Québec (two PTC, four level I,four level II, 20 level III and 28 level IV ATC)	Children **< 17 yoa** with **moderate to severe injuries** (LOS>48hrs, transfer-in, ICU unit admission, or death)	PTC; **I**; MoH	PTC; **I**; MoH<br>PTC; **II**; MoH<br>PTC; **III**; MoH<br>PTC; **IV**; MoH	Mortality*
Potoka 2002[50]	USA 1993-1997	Trauma centers in Pennsylvania (PTOS)	Children **<17 yoa** with **blunt splenic injuries**	PTC; **I**; state ± ACS	ATC; **I-II**; state ± ACS	Splenectomy*
Osler 2001[51]	USA 1985-1996	Trauma centers submitting to the National Pediatric Trauma Registry	Children **<19 yoa** admitted for **injury**	PTC; nr; state ± ACS	CTC; nr; state ± ACS	Mortality*
Potoka, 2001[52]	USA 1993-1997	All trauma centers in Pennsylvania	Children **0 to 16 yoa** admitted for injury	PTC; **I**; PTSF	ATC; **I**; PTSF<br>ATC; **II**; PTSF<br>CTC; **I**; PTSF	Functional status on discharge
Potoka, 2000[53]	USA 1993-1997	All trauma centers in Pennsylvania	Children **0 to 16 yoa** admitted for injury	PTC; **I**; PTSF	ATC; **I**; PTSF<br>ATC; **II**; PTSF<br>CTC; **I**; PTSF	Mortality
Frumiento, 2000[54]	USA 1985-1995	All hospitals in Vermont	Children **< 19 yoa** admitted for **splenic injury**	PTC; **I**; nr	Regional hospitals	Hospital charges<br>Nonoperative management
Bernardo, 1997[55]	USA 1993	University-affiliated PTCs and ATCs in Pennsylvania	Adolescents **14-18 yoa** admitted for ≥48h with **isolated musculoskeletal, chest, or abdominal injuries**	PTC; level **I**; PTSF	ATC; level **I**; PTSF	Prescription and administration of analgesics
Nakayama, 1992[56]	USA 1986-1989	All trauma centers in Pennsylvania	Children **< 15 yoa** admitted for injury	PTC; nr; PTSF	ATC; nr; PTSF	Mortality

© 2023 Moore L et al. *JAMA Network Open.*

ACS, American College of Surgeons; ATC, adult trauma center; CT, computed tomography; CTC, Combined adult and pediatric trauma centers; FSD, functional status at discharge; ICU, intensive care unit; LOS, length of stay; MRI, magnetic resonance imaging; NTDB, National Trauma Data Bank; PTC, pediatric trauma center; PTSF, Pennsylvania State Trauma Systems Foundation; TQIP, Trauma Quality Improvement Program; USA, United States of America; Yoa, years of age

*Comparisons minimally adjusted for age and injury severity

References from studies are available in eReferences 3 below

© 2023 Moore L et al. *JAMA Network Open.*

eReferences 3 – References of Included Studies

1. Gerber N, Munnangi S, Vengalasetti Y, et al. Trauma center variation of head computed tomography utilization in children presenting with mild traumatic brain injury. *Clin Imaging* 2023;94:125-31.
2. Derderian SC, Meier M, Bensard DD, et al. Adolescent blunt solid organ injury: Differences in management strategies and outcomes between pediatric and adult trauma centers. *Am J Surg* 2022.
3. Hairr M, Tumin D, Greene E, et al. Facility Size and Risk Factors for Mortality in Pediatric Trauma. *Am Surg* 2022:31348221121555.
4. Lewit RA, Veras LV, Kocak M, et al. Pediatric traumatic brain injury: Resource utilization and outcomes at adult versus pediatric trauma centers. *Surgery Open Science* 2022;7:68-73.
5. Killien EY, Grassia KL, Butler EK, et al. Variation in Tracheostomy Placement and Outcomes following Pediatric Trauma among Adult, Pediatric, and Combined Trauma Centers. *J Trauma Acute Care Surg* 2022.
6. Pulido OR, Morgan ME, Bradburn E, et al. A Statewide Analysis of Pediatric Liver Injuries Treated at Adult Versus Pediatric Trauma Centers. *Journal of Surgical Research* 2022;272:184-9.
7. Sheff ZT, Engbrecht BW, Rodgers R, et al. Mortality of adolescents with isolated traumatic brain injury does not vary with type of level I trauma center. *J Trauma Acute Care Surg* 2022;93:538-44.
8. Stephenson KJ, Shewmake CN, Bowman SM, et al. Elder child or young adult? Adolescent trauma mortality amongst pediatric and adult facilities. *Am J Surg* 2022;224:1445-9.
9. Ali A, Tatum D, Jones G, et al. Computed Tomography for Pediatric Pelvic Fractures in Pediatric Versus Adult Trauma Centers. *Journal of Surgical Research* 2021;259:47-54.
10. Evans J, Murch H, Begley R, et al. Mortality in adolescent trauma: a comparison of children's, mixed and adult major trauma centres. *Emergency Medicine Journal* 2021;38:488-94.
11. Khalil M, Alawwa G, Pinto F, et al. Pediatric mortality at pediatric versus adult trauma centers. *Journal of Emergencies Trauma and Shock* 2021;14:128-35.
12. Kim W, Ahn N, Ata A, et al. Pediatric cervical spine injury in the United States: Defining the burden of injury, need for operative intervention, and disparities in imaging across trauma centers. *J Pediatr Surg* 2021;56:293-6.
13. Massoumi R, Wertz J, Duong T, et al. Variation in pediatric cervical spine imaging across trauma centers-A cause for concern? *J Trauma Acute Care Surg* 2021;91:641-8.
14. Scantling D, Hatchimonji J, Williamson J, et al. Adjacent Adult and Pediatric Trauma Centers: Which Way to Turn With the Injured Adolescent? *Am Surg* 2021:31348211033539.
15. Swendiman RA, Luks VL, Hatchimonji JS, et al. Mortality After Adolescent Firearm Injury: Effect of Trauma Center Designation. *Journal of Adolescent Health* 2021;68:978-84.
16. Swendiman RA, Abramov A, Fenton SJ, et al. Use of angioembolization in pediatric polytrauma patients WITH BLUNT SPLENIC INJURYAngioembolization in Pediatric Blunt Splenic Injury. *Journal of Pediatric Surgery* 2021;56:2045-51.
17. Yanchar NL, Lockyer L, Ball CG, et al. Pediatric versus adult paradigms for management of adolescent injuries within a regional trauma system. *Journal of Pediatric Surgery* 2021;56:512-9.
18. Filipescu R, Powers C, Yu H, et al. The adherence of adult trauma centers to American Pediatric Surgical Association guidelines on management of blunt splenic injuries. *Journal of Pediatric Surgery* 2020;55:1748-53.
19. Hatchimonji JS, Swendiman RA, Goldshore MA, et al. Pediatric firearm mortality in the United States, 2010 to 2016: A National Trauma Data Bank analysis. *Journal of Trauma and Acute Care Surgery* 2020;88:402-7.
20. Prieto JM, Van Gent JM, Calvo RY, et al. Pediatric extremity vascular trauma: It matters where it is treated. *Journal of Trauma and Acute Care Surgery* 2020;88:469-76.
21. Prieto JM, Van Gent JM, Calvo RY, et al. Evaluating surgical outcomes in pediatric extremity vascular trauma. *J Pediatr Surg* 2020;55:319-23.
22. Strait L, Sussman R, Ata A, et al. Utilization of CT imaging in minor pediatric head, thoracic, and abdominal trauma in the United States. *J Pediatr Surg* 2020;55:1766-72.
23. Swendiman RA, Goldshore MA, Fenton SJ, et al. Defining the role of angioembolization in pediatric isolated blunt solid organ injury. *Journal of Pediatric Surgery* 2020;55:688-92.
24. Yung N, Solomon D, Schuster K, et al. Closing the gap in care of blunt solid organ injury in children. *J Trauma Acute Care Surg* 2020;89:894-9.

© 2023 Moore L et al. *JAMA Network Open.*

25. Alexander M, Zaghal A, Wetjen K, et al. Pediatric trauma center verification improves quality of care and reduces resource utilization in blunt splenic injury. *Journal of Pediatric Surgery* 2019;54:155-9.
26. Myers SR, Branas CC, French B, et al. A National Analysis of Pediatric Trauma Care Utilization and Outcomes in the United States. *Pediatric Emergency Care* 2019;35:1-7.
27. Sathya C, Alali AS, Wales PW, et al. Computed tomography rates and estimated radiation-associated cancer risk among injured children treated at different trauma center types. *Injury* 2019;50:142-8.
28. Vassallo J, Webster M, Barnard EBG, et al. Epidemiology and aetiology of paediatric traumatic cardiac arrest in England and Wales. *Arch Dis Child* 2019;104:437-43.
29. Bardes JM, Benjamin E, Escalante AA, et al. Severe traumatic brain injuries in children: Does the type of trauma center matter? *J Pediatr Surg* 2018;53:1523-5.
30. Schlegel C, Greeno A, Chen H, et al. Evolution of a level I pediatric trauma center: Changes in injury mechanisms and improved outcomes. *Surgery* 2018;163:1173-7.
31. Adams SE, Holland A, Brown J. Management of paediatric splenic injury in the New South Wales trauma system. *Injury-Int J Care Inj* 2017;48:106-13.
32. Mitchell RJ, Curtis K, Testa L, et al. Differences in survival outcome for severely injured paediatric trauma by type of trauma centre. *J Paediatr Child Health* 2017;53:808-13.
33. Miyata S, Cho J, Matsushima K, et al. Association between pediatric blunt splenic injury volume and the splenectomy rate. *Journal of Pediatric Surgery* 2017;52:1816-21.
34. Miyata S, Cho J, Lebedevskiy O, et al. Trauma experts versus pediatric experts: comparison of outcomes in pediatric penetrating injuries. *Journal of Surgical Research* 2017;208:173-9.
35. Baudin F, Floccard B, Desgranges FP, et al. [Effectiveness of management of children with severe trauma in a pediatric trauma center and in an adult trauma center: A before-after study]. *Arch Pediatr* 2016;23:367-72.
36. Pandit V, Michailidou M, Rhee P, et al. The use of whole body computed tomography scans in pediatric trauma patients: Are there differences among adults and pediatric centers? *Journal of Pediatric Surgery* 2016;51:649-53.
37. Safavi A, Skarsgard ED, Rhee P, et al. Trauma center variation in the management of pediatric patients with blunt abdominal solid organ injury: a national trauma data bank analysis. *Journal of Pediatric Surgery* 2016;51:499-502.
38. Walther AE, Falcone RA, Pritts TA, et al. Pediatric and adult trauma centers differ in evaluation, treatment, and outcomes for severely injured adolescents. *J Pediatr Surg* 2016;51:1346-50.
39. Webman RB, Carter EA, Mittal S, et al. Association Between Trauma Center Type and Mortality Among Injured Adolescent Patients. *JAMA Pediatr* 2016;170:780-6.
40. Kelley-Quon LI, Crowley MA, Applebaum H, et al. Academic-community partnerships improve outcomes in pediatric trauma care. *Journal of Pediatric Surgery* 2015;50:1032-6.
41. Marin JR, Wang L, Winger DG, et al. Variation in Computed Tomography Imaging for Pediatric Injury-Related Emergency Visits. *J Pediatr* 2015;167:897-904.e3.
42. Sathya C, Alali AS, Wales PW, et al. Mortality Among Injured Children Treated at Different Trauma Center Types. *JAMA Surgery* 2015;150:874-81.
43. Walther AE, Pritts TA, Falcone RA, et al. Teen trauma without the drama: outcomes of adolescents treated at Ohio adult versus pediatric trauma centers. *J Trauma Acute Care Surg* 2014;77:109-16; discussion 16.
44. Lippert SJ, Hartin CW, Ozgediz DE, et al. Splenic conservation: Variation between pediatric and adult trauma centers. *Journal of Surgical Research* 2013;182:17-20.
45. Matsushima K, Schaefer EW, Won EJ, et al. Injured Adolescents, Not Just Large Children: Difference in Care and Outcome between Adult and Pediatric Trauma Centers. *American Surgeon* 2013;79:267-73.
46. Matsushima K, Kulaylat AN, Won EJ, et al. Variation in the management of adolescent patients with blunt abdominal solid organ injury between adult versus pediatric trauma centers: an analysis of a statewide trauma database. *Journal of Surgical Research* 2013;183:808-13.
47. Mitchell RJ, Curtis K, Chong S, et al. Comparative analysis of trends in paediatric trauma outcomes in New South Wales, Australia. *Injury* 2013;44:97-103.
48. Wang NE, Saynina O, Vogel LD, et al. The effect of trauma center care on pediatric injury mortality in California, 1999 to 2011. *Journal of Trauma & Acute Care Surgery* 2013;75:704-16.
49. Amini R, Lavoie A, Moore L, et al. Pediatric trauma mortality by type of designated hospital in a mature inclusive trauma system. *J Emerg Trauma Shock* 2011;4:12-9.

© 2023 Moore L et al. *JAMA Network Open.*

50. Potoka DA, Schall LC, Ford HR. Risk factors for splenectomy in children with blunt splenic trauma. *J Pediatr Surg* 2002;37:294-9.
51. Osler TM, Vane DW, Tepas JJ, et al. Do pediatric trauma centers have better survival rates than adult trauma centers? An examination of the rational pediatric trauma registry. *Journal of Trauma-Injury Infection and Critical Care* 2001;50:96-9.
52. Potoka DA, Schall LC, Ford HR. Improved functional outcome for severely injured children treated at pediatric trauma centers. *Journal of Trauma-Injury Infection and Critical Care* 2001;51:824-32.
53. Potoka DA, Schall LC, Gardner MJ, et al. Impact of pediatric trauma centers on mortality in a statewide system. *J Trauma* 2000;49:237-45.
54. Frumiento C, Vane DW. Changing patterns of treatment for blunt splenic injuries: an 11-year experience in a rural state. *J Pediatr Surg* 2000;35:985-8; discussion 8-9.
55. Bernardo LM, Gardner MJ, Miracle TB, et al. Analgesic prescription and administration to adolescents admitted to an adult versus pediatric trauma center. *J Trauma Nurs* 1997;4:13-21.
56. Nakayama DK, Copes WS, Sacco W. Differences in trauma care among pediatric and nonpediatric trauma centers. *J Pediatr Surg* 1992;27:427-31.

© 2023 Moore L et al. *JAMA Network Open.*

eFigure 2. Enhanced Funnel Plot of Studies Evaluating the Odds of mortality in Pediatric Major Trauma at Pediatric Trauma Centers vs Adult Trauma Centers (Trim and Fill Method)

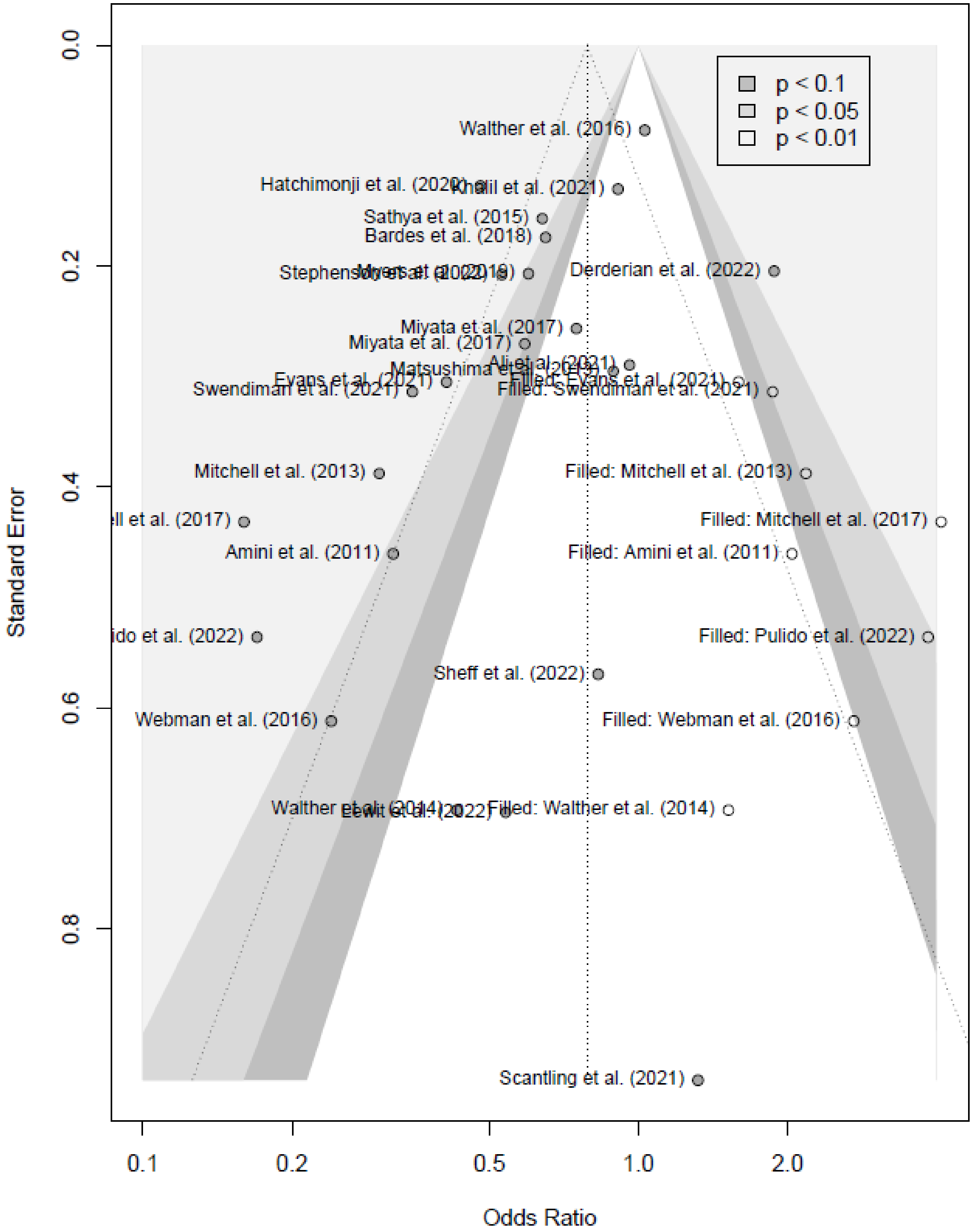

© 2023 Moore L et al. *JAMA Network Open.*

eFigure 3. Forest Plots Describing the Odds of Mortality for Children Aged 15 Years or Less Admitted to Hospitals Following Trauma Treated at Pediatric Trauma Centers (PTCs) Versus Adult Trauma Centers (ATCs)

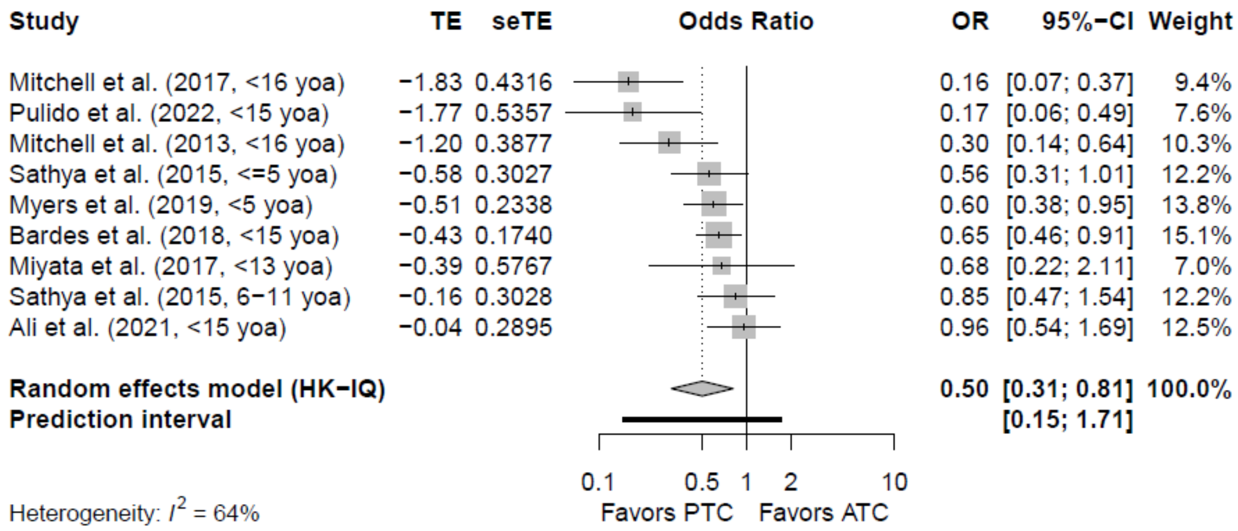

© 2023 Moore L et al. *JAMA Network Open.*

eFigure 4. Forest Plots Describing the Odds of Mortality for Children Aged 14 Years or Less Admitted to Hospitals Following Trauma Treated at pediatric Trauma Centers (PTCs) Versus Adult Trauma Centers (ATCs)

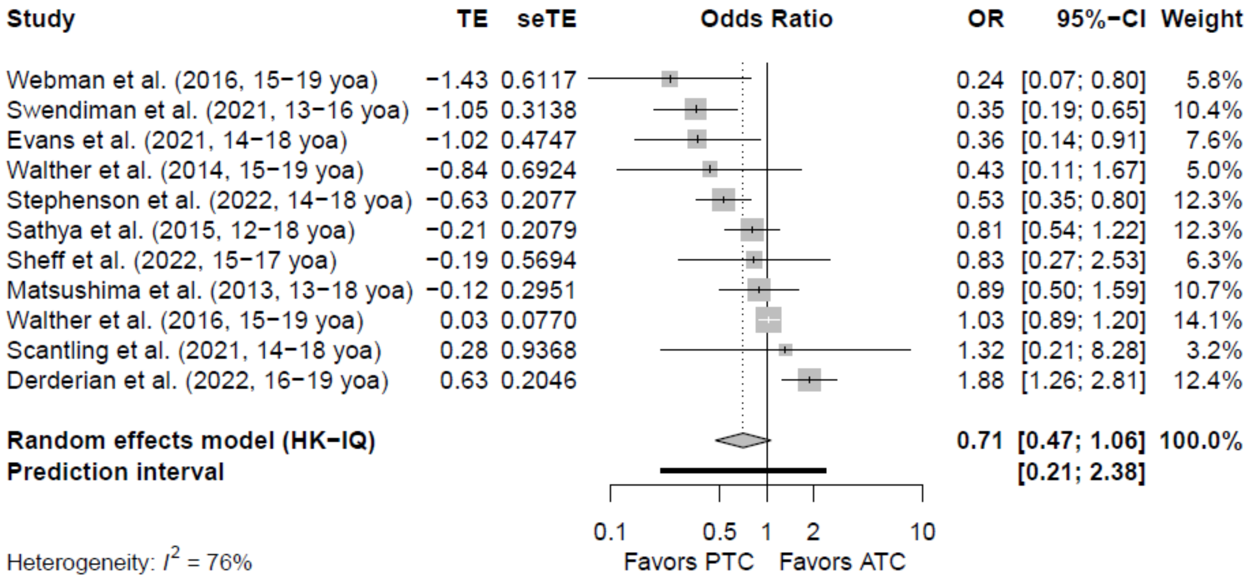

© 2023 Moore L et al. *JAMA Network Open.*

eFigure 5. Forest Plots Describing the Odds of Mortality for Children With Head Injury Treated at Pediatric Trauma Centers (PTCs) vs Adult Trauma Centers (ATCs)

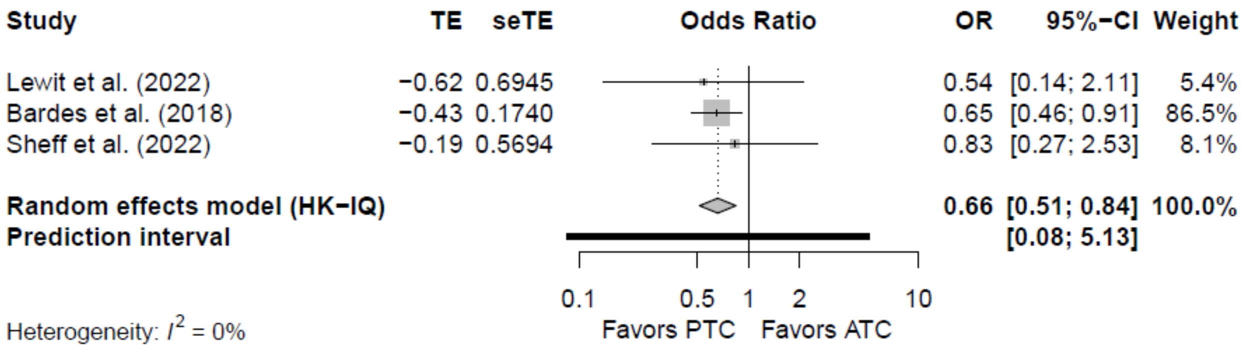

© 2023 Moore L et al. *JAMA Network Open.*

eFigure 6. Forest Plots Describing the Odds of Mortality for Children With Blunt Solid Organ Injury Treated at Pediatric Trauma Centers (PTCs) vs Adult Trauma Centers (ATCs)

Study	TE	seTE	OR	95%−CI	Weight
Pulido et al. (2022)	−1.77	0.5357	0.17	[0.06; 0.49]	29.8%
Miyata et al. (2017)	−0.29	0.2565	0.75	[0.45; 1.24]	34.8%
Derderian et al. (2022)	0.63	0.2046	1.88	[1.26; 2.81]	35.4%
**Random effects model (HK−IQ)**			**0.67**	**[0.03; 12.79]**	**100.0%**
**Prediction interval**				**[0.00; 10159707.79]**	

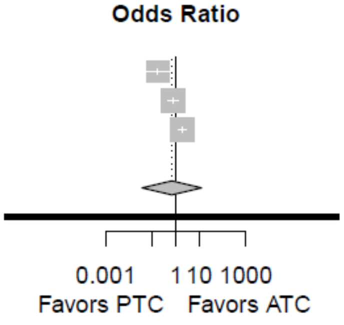

Heterogeneity: $I^2 = 91\%$

© 2023 Moore L et al. *JAMA Network Open.*

eFigure 7. Forest Plots Describing the Odds of Mortality for Children With Penetrating Trauma Treated at Pediatric Trauma Centers (PTCs) vs Adult Trauma Centers (ATCs)

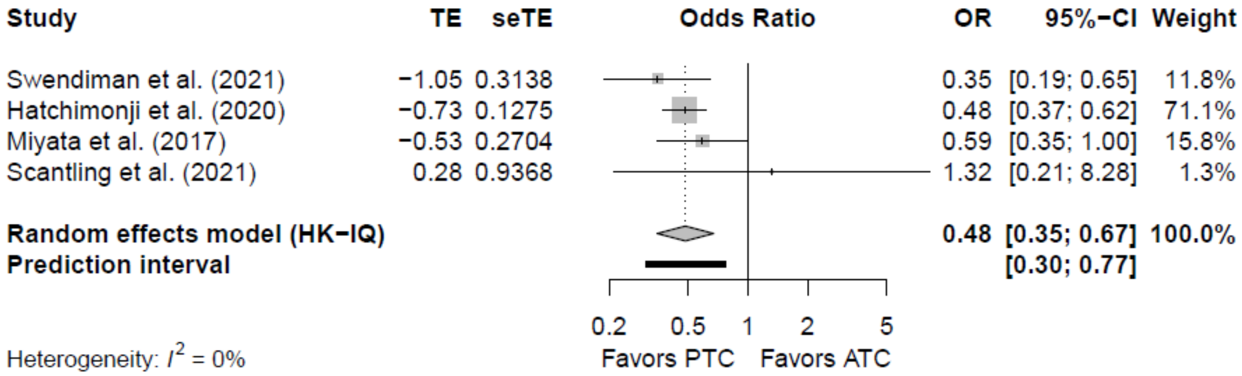

© 2023 Moore L et al. *JAMA Network Open.*

eFigure 8. Forest Plots Describing the Odds of Mortality for Children With Major Trauma (ISS ≥ 12) Treated at Pediatric Trauma Centers (PTCs) vs Adult Trauma Centers (ATCs)

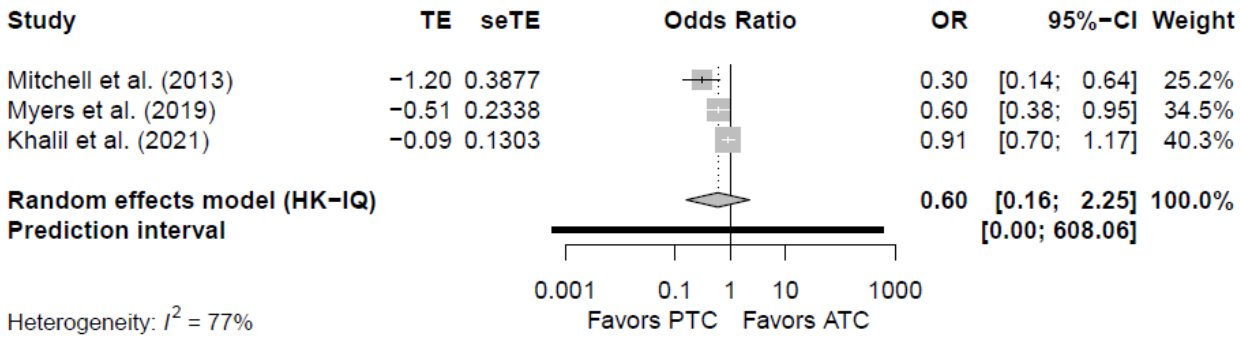

© 2023 Moore L et al. *JAMA Network Open.*

eFigure 9. Forest Plots Describing the Odds of Mortality for children Admitted to Hospitals Following Trauma Treated at Level I Pediatric Trauma Centers (PTCs) vs Level I Adult Trauma Centers (ATCs)

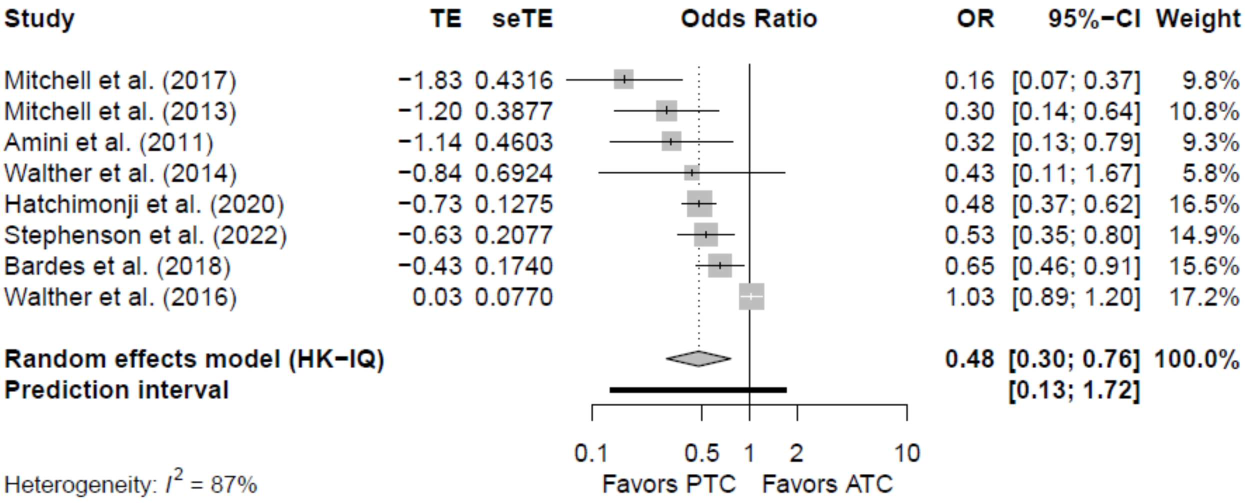

© 2023 Moore L et al. *JAMA Network Open.*

eFigure 10. Forest Plots Describing the Odds of Mortality for Children Admitted to Hospitals Following Trauma Treated at Level I and II Pediatric Trauma Centers (PTCs) vs Level I and II Adult Trauma Centers (ATCs)

Study	TE	seTE	OR	95%−CI	Weight
Pulido et al. (2022)	−1.77	0.5357	0.17	[0.06; 0.49]	3.9%
Webman et al. (2016)	−1.43	0.6117	0.24	[0.07; 0.80]	3.1%
Swendiman et al. (2021)	−1.05	0.3138	0.35	[0.19; 0.65]	9.4%
Evans et al. (2021)	−1.02	0.4747	0.36	[0.14; 0.91]	4.8%
Stephenson et al. (2022)	−0.63	0.2077	0.53	[0.35; 0.80]	15.9%
Myers et al. (2019)	−0.51	0.2069	0.60	[0.40; 0.90]	15.9%
Sathya et al. (2015)	−0.45	0.1571	0.64	[0.47; 0.87]	20.7%
Miyata et al. (2017)	−0.29	0.2565	0.75	[0.45; 1.24]	12.3%
Sheff et al. (2022)	−0.19	0.5694	0.83	[0.27; 2.53]	3.5%
Ali et al. (2021)	−0.04	0.2895	0.96	[0.54; 1.69]	10.5%
**Random effects model (HK−IQ)**			**0.56**	**[0.41; 0.76]**	**100.0%**
**Prediction interval**				**[0.34; 0.93]**	

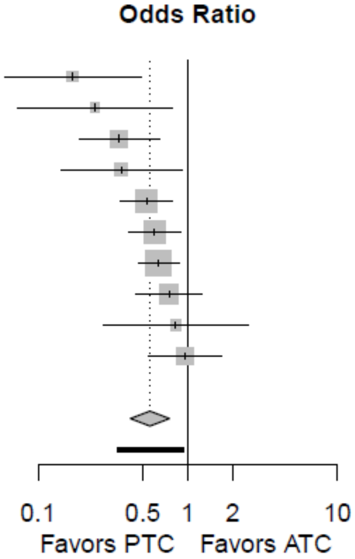

Heterogeneity: $I^2 = 44\%$

© 2023 Moore L et al. *JAMA Network Open.*

eFigure 11. Forest Plots Describing the Odds of Mortality for children Admitted to Hospitals Following Trauma Treated at Level I and II Pediatric Trauma Centers (PTCs) vs Level I and II Adult Trauma Centers (ATCs) With the Same Verification Organization

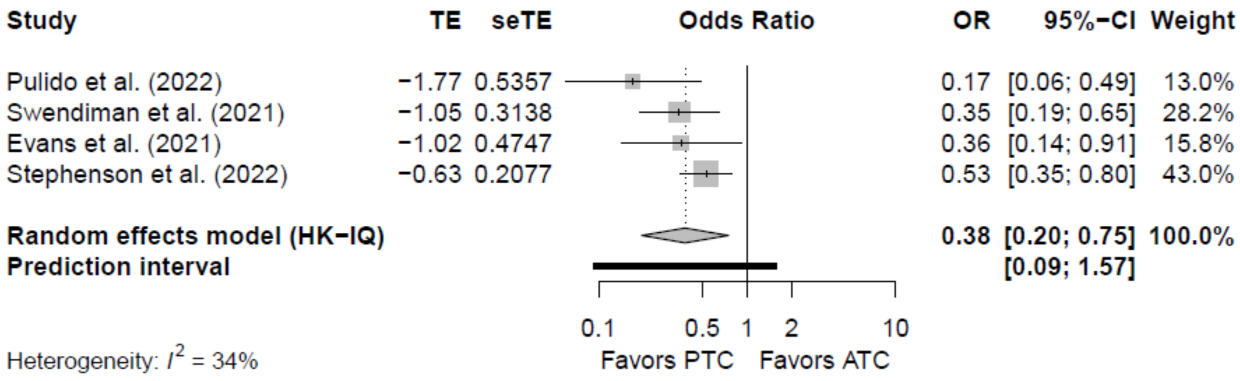

© 2023 Moore L et al. *JAMA Network Open.*

eFigure 12. Forest Plots Describing the Odds of Mortality for Children Admitted to Hospitals Following Trauma Treated at Pediatric Trauma Centers (PTCs) vs Adult Trauma Centers (ATCs) in the US

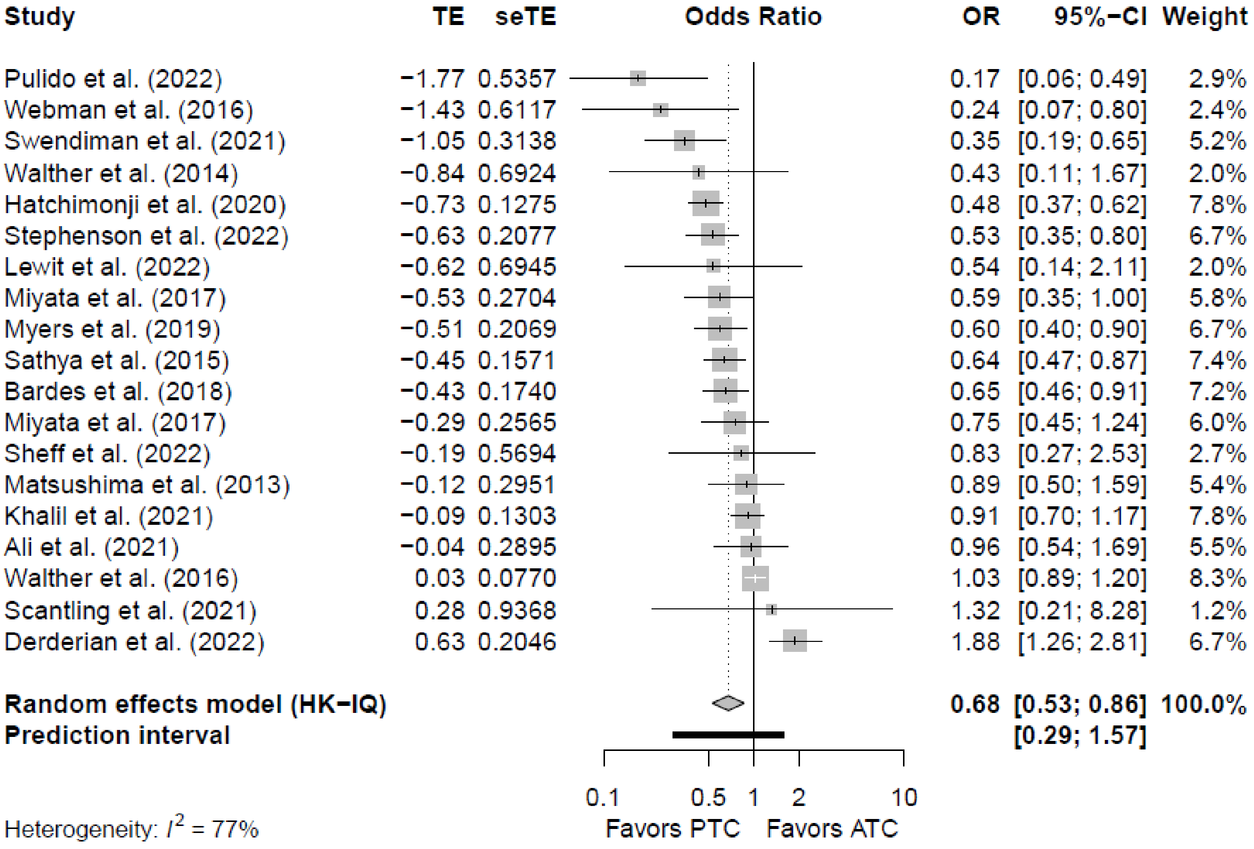

© 2023 Moore L et al. *JAMA Network Open.*

eFigure 13. Forest Plots Describing the Odds of Mortality for Children Admitted to Hospitals Following Trauma Treated at Pediatric Trauma Centers (PTCs) vs Adult Trauma Centers (ATCs) in Other Countries

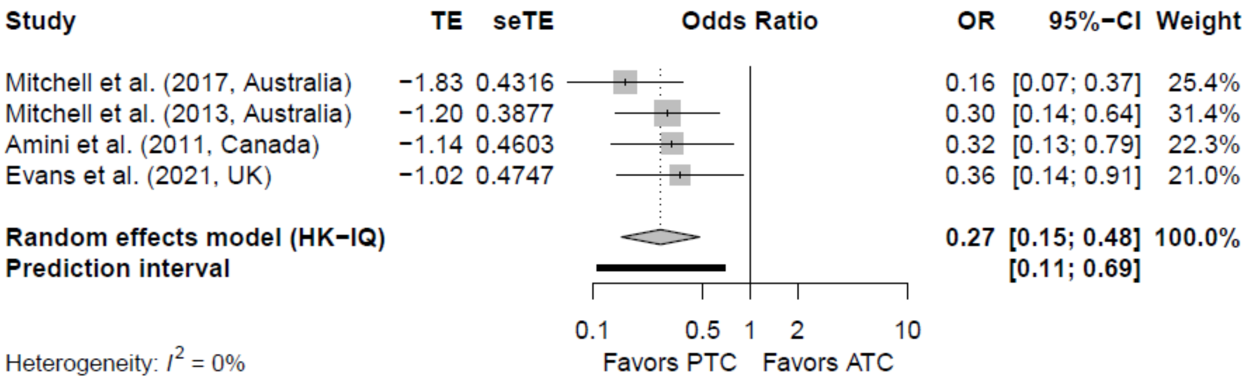

© 2023 Moore L et al. *JAMA Network Open.*

eFigure 14. Forest Plots Describing the Odds of Mortality for Children Admitted to Hospitals Following Trauma Treated at Pediatric Trauma Centers (PTCs) vs Adult Trauma Centers (ATCs) in Studies Recruiting Patients up to 2010 to 2014

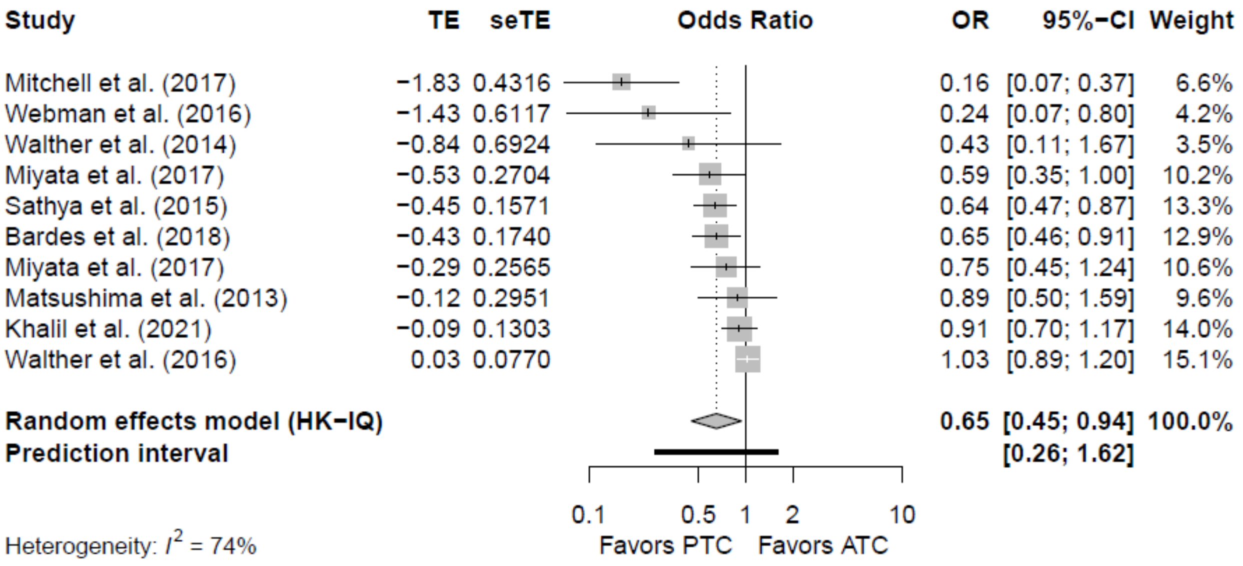

© 2023 Moore L et al. *JAMA Network Open.*

eFigure 15. Forest Plots Describing the Odds of Mortality for Children Admitted to Hospitals Following Trauma Treated at Pediatric Trauma Centers (PTCs) vs Adult Trauma Centers (ATCs) in Studies Recruiting Patients up to 2015 to 2023

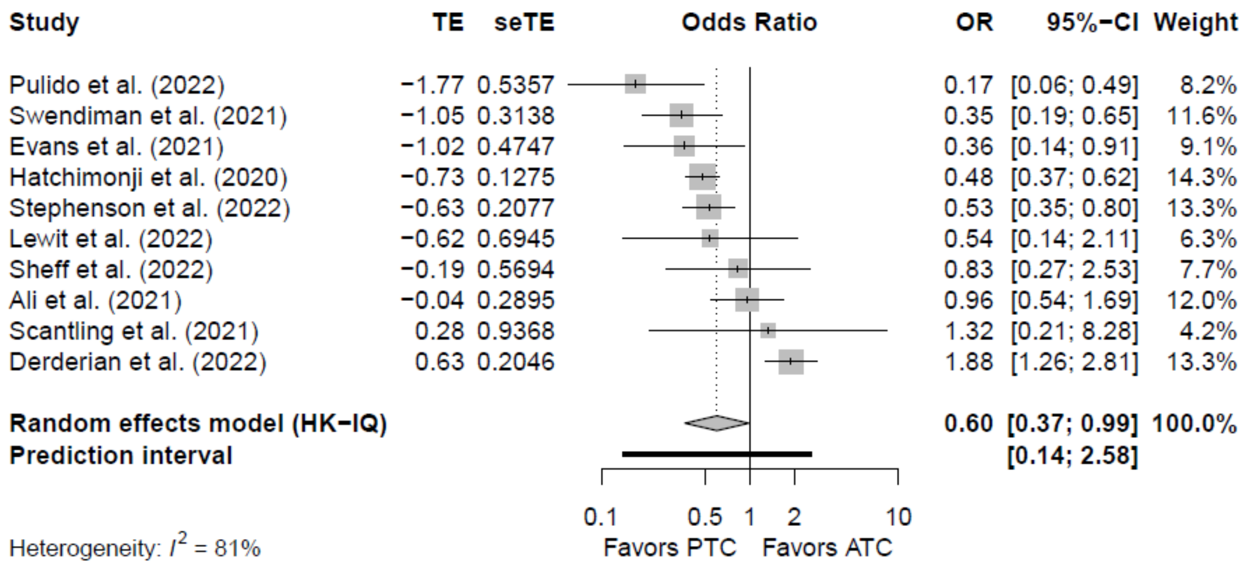

© 2023 Moore L et al. *JAMA Network Open.*

eFigure 16. Forest Plots Describing the Odds of Mortality for Children Admitted to Hospitals Following Trauma Treated at Pediatric Trauma Centers (PTCs) vs Adult Trauma Centers (ATCs) With Studies at Moderate (n=1) or Serious Risk of Bias

Study	TE	seTE	OR	95%−CI	Weight
Mitchell et al. (2017)	−1.83	0.4316	0.16	[0.07; 0.37]	4.4%
Pulido et al. (2022)	−1.77	0.5357	0.17	[0.06; 0.49]	3.4%
Webman et al. (2016)	−1.43	0.6117	0.24	[0.07; 0.80]	2.8%
Mitchell et al. (2013)	−1.20	0.3877	0.30	[0.14; 0.64]	4.9%
Amini et al. (2011)	−1.14	0.4603	0.32	[0.13; 0.79]	4.1%
Evans et al. (2021)	−1.02	0.4747	0.36	[0.14; 0.91]	3.9%
Walther et al. (2014)	−0.84	0.6924	0.43	[0.11; 1.67]	2.3%
Hatchimonji et al. (2020)	−0.73	0.1275	0.48	[0.37; 0.62]	9.0%
Stephenson et al. (2022)	−0.63	0.2077	0.53	[0.35; 0.80]	7.7%
Lewit et al. (2022)	−0.62	0.6945	0.54	[0.14; 2.11]	2.3%
Miyata et al. (2017)	−0.53	0.2704	0.59	[0.35; 1.00]	6.7%
Myers et al. (2019)	−0.51	0.2069	0.60	[0.40; 0.90]	7.7%
Sathya et al. (2015)	−0.45	0.1571	0.64	[0.47; 0.87]	8.6%
Bardes et al. (2018)	−0.43	0.1740	0.65	[0.46; 0.91]	8.3%
Matsushima et al. (2013)	−0.12	0.2951	0.89	[0.50; 1.59]	6.3%
Ali et al. (2021)	−0.04	0.2895	0.96	[0.54; 1.69]	6.4%
Walther et al. (2016)	0.03	0.0770	1.03	[0.89; 1.20]	9.6%
Scantling et al. (2021)	0.28	0.9368	1.32	[0.21; 8.28]	1.4%
**Random effects model (HK−IQ)**			**0.53**	**[0.41; 0.69]**	**100.0%**
**Prediction interval**				**[0.23; 1.25]**	

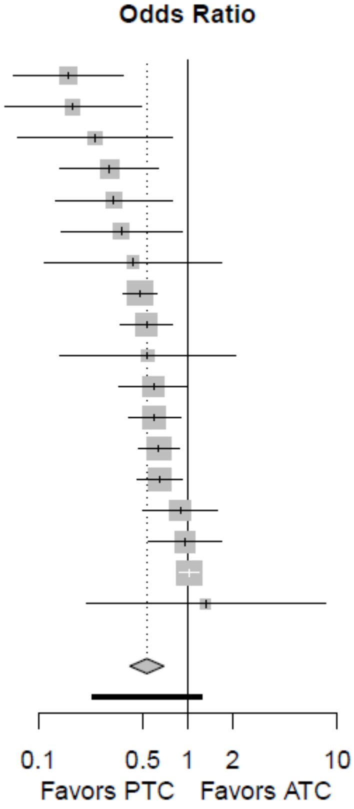

Heterogeneity: $I^2 = 76\%$

© 2023 Moore L et al. *JAMA Network Open.*

eFigure 17. Forest Plots Describing the Odds of Mortality for Children Admitted to Hospitals Following Trauma Treated at Pediatric Trauma Centers (PTCs) vs Adult Trauma Centers (ATCs) With Studies at Critical Risk of Bias

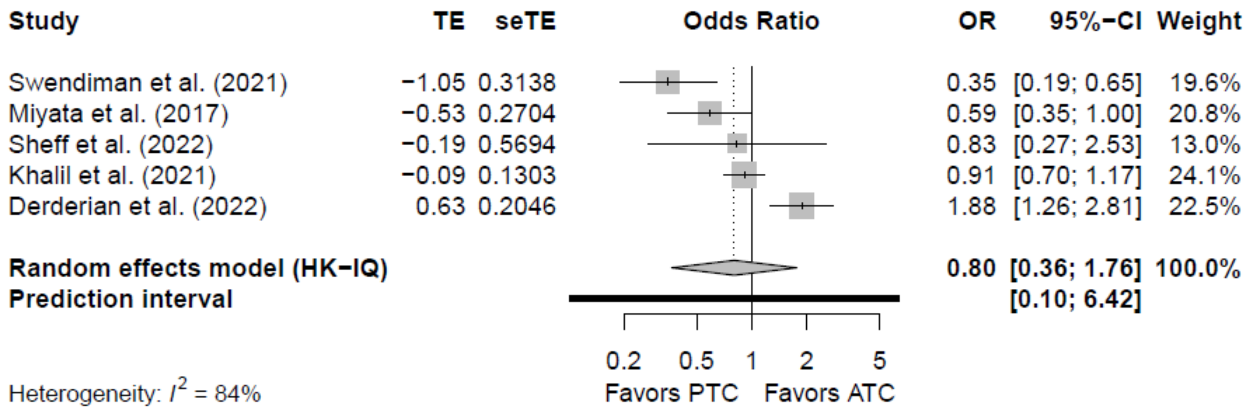

© 2023 Moore L et al. *JAMA Network Open.*

eFigure 18. Forest Plots Describing the Odds of Mortality for Children Admitted to Hospitals Following Trauma Treated at Pediatric Trauma Centers (PTCs) vs Adult Trauma Centers (ATCs) for Studies With Transfers Excluded

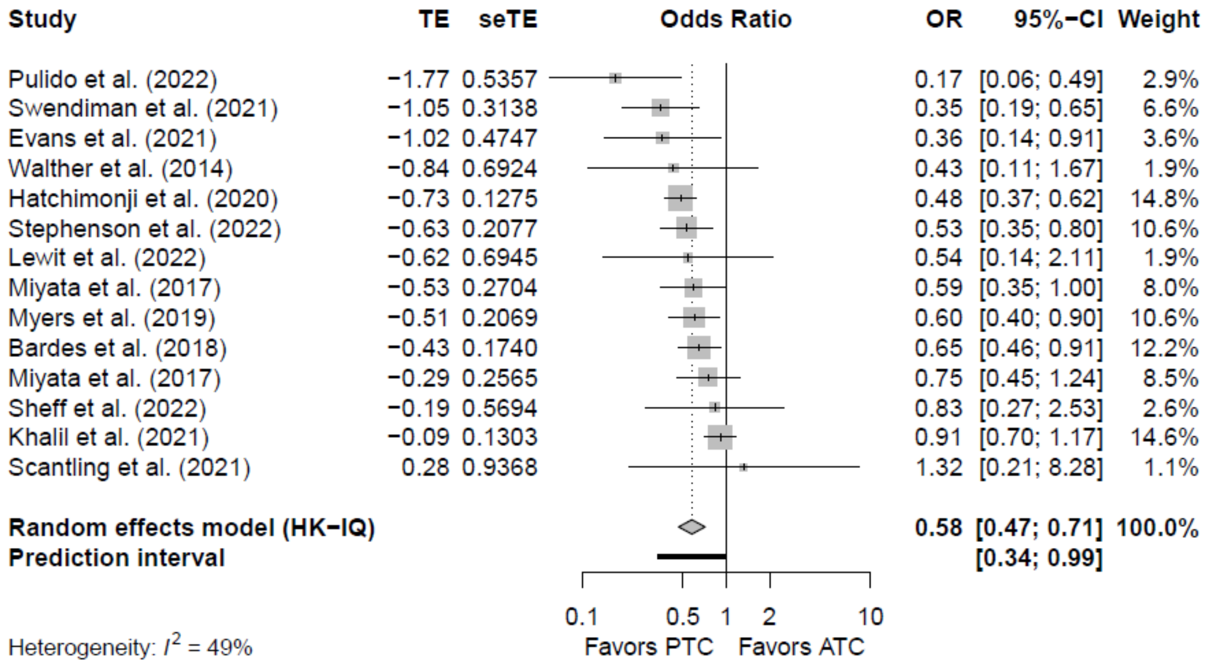

© 2023 Moore L et al. *JAMA Network Open.*

eFigure 19. Forest Plots Describing the Odds of Mortality for Children Admitted to Hospitals Following Trauma Treated at Pediatric Trauma Centers (PTCs) vs Adult Trauma Centers (ATCs) for Studies With Transfers Included and Adjusted For

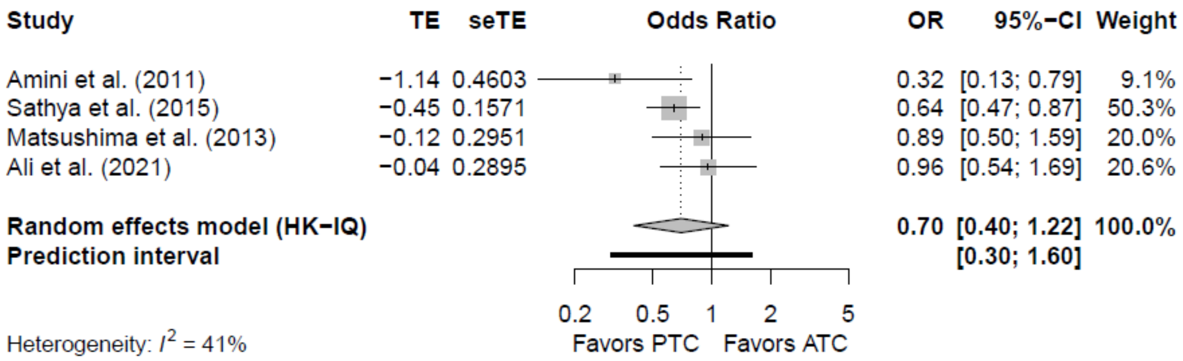

© 2023 Moore L et al. *JAMA Network Open.*

eFigure 20. Forest Plots Describing the Odds of Mortality for Children Admitted to Hospitals Following Trauma Treated at Pediatric Trauma Centers (PTCs) vs Adult Trauma Centers (ATCs) for Studies With Transfers Included and Not Adjusted For

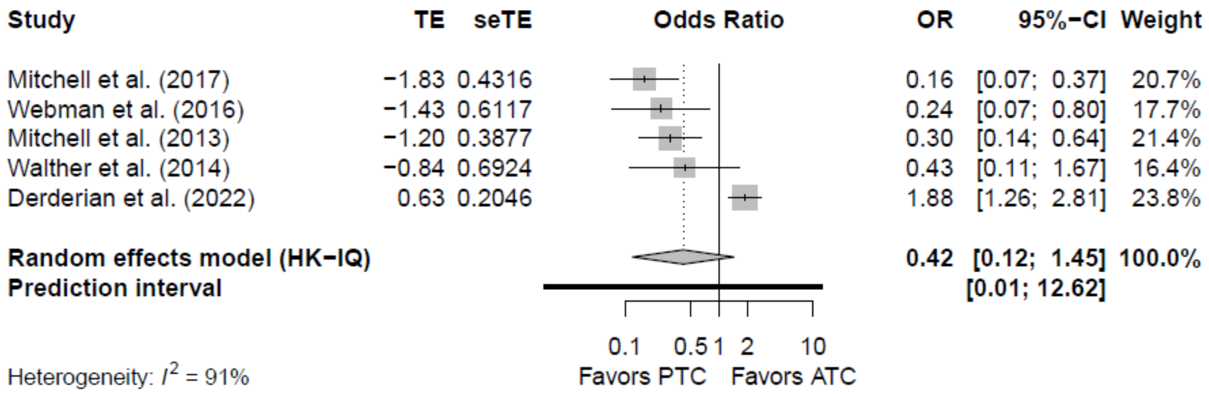

© 2023 Moore L et al. *JAMA Network Open.*

eFigure 21. Forest Plots Describing the Odds of Mortality for Children Admitted to Hospitals Following Trauma Treated at Pediatric Trauma Centers (PTCs) vs Adult Trauma Centers (ATCs) With 7 Outliers Removed

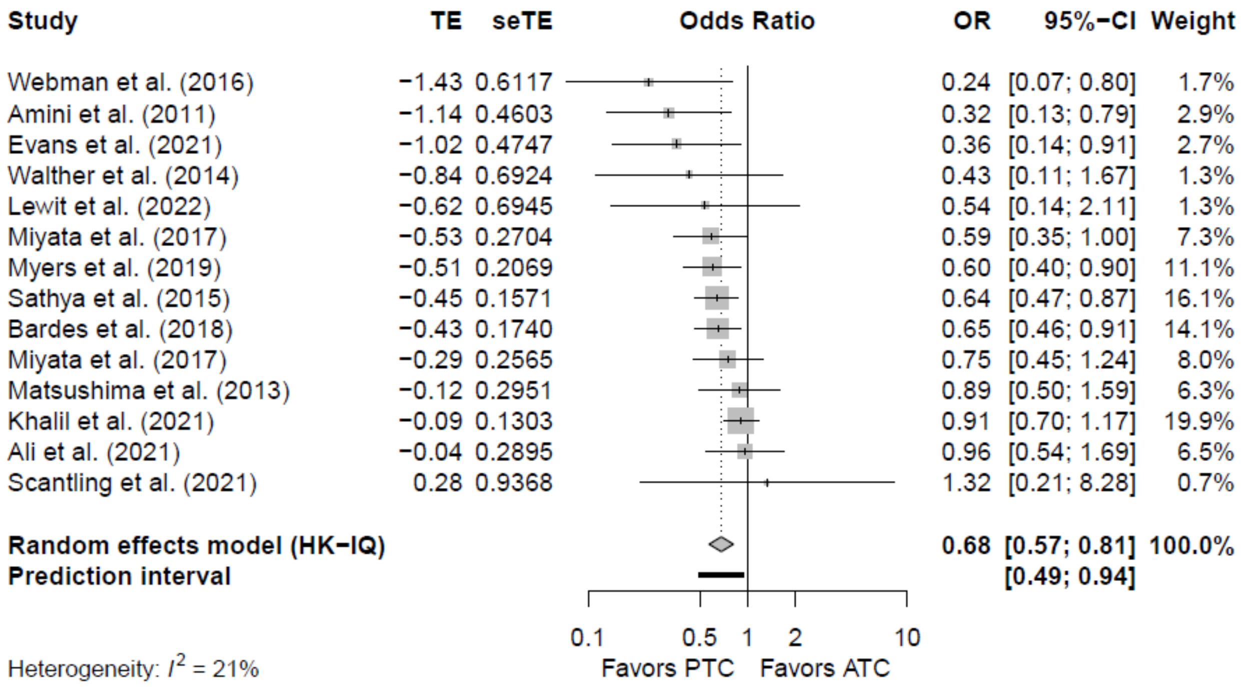

© 2023 Moore L et al. *JAMA Network Open.*

eFigure 22. Forest Plots Describing the Odds of Mortality for Children Admitted to Hospitals Following Trauma Treated at Pediatric Trauma Centers (PTCs) vs Adult Trauma Centers (ATCs) With 3 Outliers Removed

Study	TE	seTE	OR	95%−CI	Weight
Pulido et al. (2022)	−1.77	0.5357	0.17	[0.06; 0.49]	2.6%
Webman et al. (2016)	−1.43	0.6117	0.24	[0.07; 0.80]	2.1%
Mitchell et al. (2013)	−1.20	0.3877	0.30	[0.14; 0.64]	4.3%
Amini et al. (2011)	−1.14	0.4603	0.32	[0.13; 0.79]	3.3%
Swendiman et al. (2021)	−1.05	0.3138	0.35	[0.19; 0.65]	5.6%
Evans et al. (2021)	−1.02	0.4747	0.36	[0.14; 0.91]	3.2%
Walther et al. (2014)	−0.84	0.6924	0.43	[0.11; 1.67]	1.7%
Hatchimonji et al. (2020)	−0.73	0.1275	0.48	[0.37; 0.62]	10.8%
Lewit et al. (2022)	−0.62	0.6945	0.54	[0.14; 2.11]	1.7%
Miyata et al. (2017)	−0.53	0.2704	0.59	[0.35; 1.00]	6.5%
Myers et al. (2019)	−0.51	0.2069	0.60	[0.40; 0.90]	8.3%
Sathya et al. (2015)	−0.45	0.1571	0.64	[0.47; 0.87]	9.9%
Bardes et al. (2018)	−0.43	0.1740	0.65	[0.46; 0.91]	9.3%
Miyata et al. (2017)	−0.29	0.2565	0.75	[0.45; 1.24]	6.9%
Matsushima et al. (2013)	−0.12	0.2951	0.89	[0.50; 1.59]	6.0%
Khalil et al. (2021)	−0.09	0.1303	0.91	[0.70; 1.17]	10.7%
Ali et al. (2021)	−0.04	0.2895	0.96	[0.54; 1.69]	6.1%
Scantling et al. (2021)	0.28	0.9368	1.32	[0.21; 8.28]	1.0%
**Random effects model (HK−IQ)**			**0.57**	**[0.46; 0.71]**	**100.0%**
**Prediction interval**				**[0.31; 1.04]**	

Odds Ratio

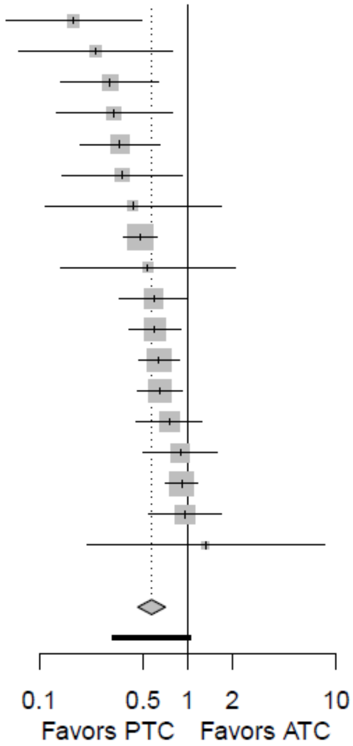

Heterogeneity: $I^2 = 54\%$

© 2023 Moore L et al. *JAMA Network Open.*

eFigure 23. Forest Plots Describing the Odds of Complications for Children Admitted to Hospitals Following Trauma Treated at pediatric Trauma Centers (PTCs) vs Adult Trauma Centers (ATCs)

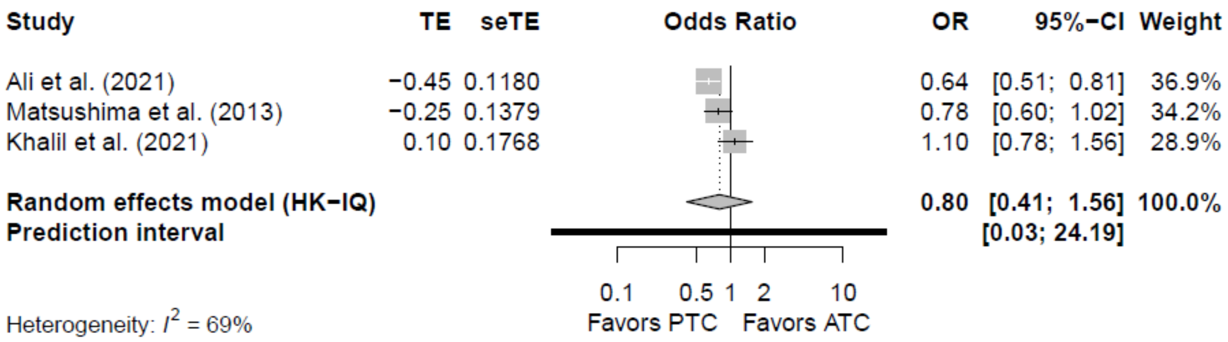

© 2023 Moore L et al. *JAMA Network Open.*

eFigure 24. Forest Plots Describing the Odds of Complications for Children Admitted to Hospitals Following Trauma Treated at Level I and II Pediatric Trauma Centers (PTCs) vs Level I and II Adult Trauma Centers (ATCs)

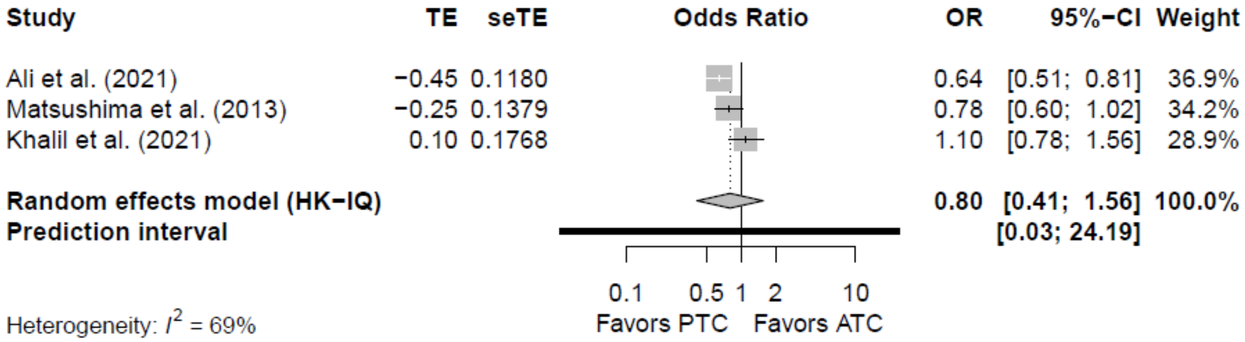

© 2023 Moore L et al. *JAMA Network Open.*

eFigure 25. Forest Plots Describing the Odds of Computed Tomography (CT) Imaging for Children Admitted to Hospitals Following Trauma Treated at Pediatric Trauma Centers (PTCs) vs Adult Trauma Centers (ATCs)

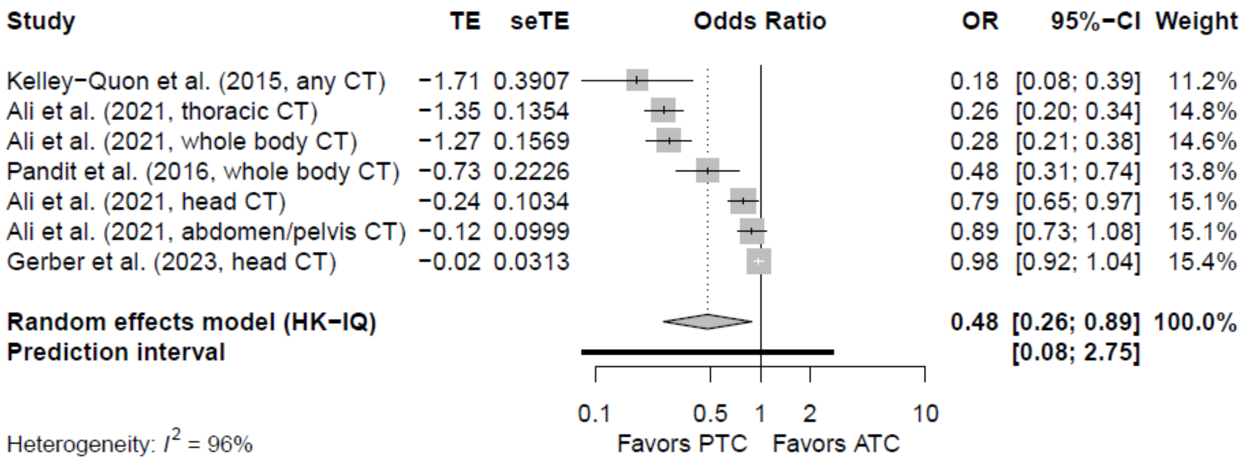

© 2023 Moore L et al. *JAMA Network Open.*

eFigure 26. Forest Plots Describing the Odds of Operative Management for Children With Blunt Solid Organ Injuries Treated at Pediatric Trauma Centers (PTCs) vs Adult Trauma Centers (ATCs)

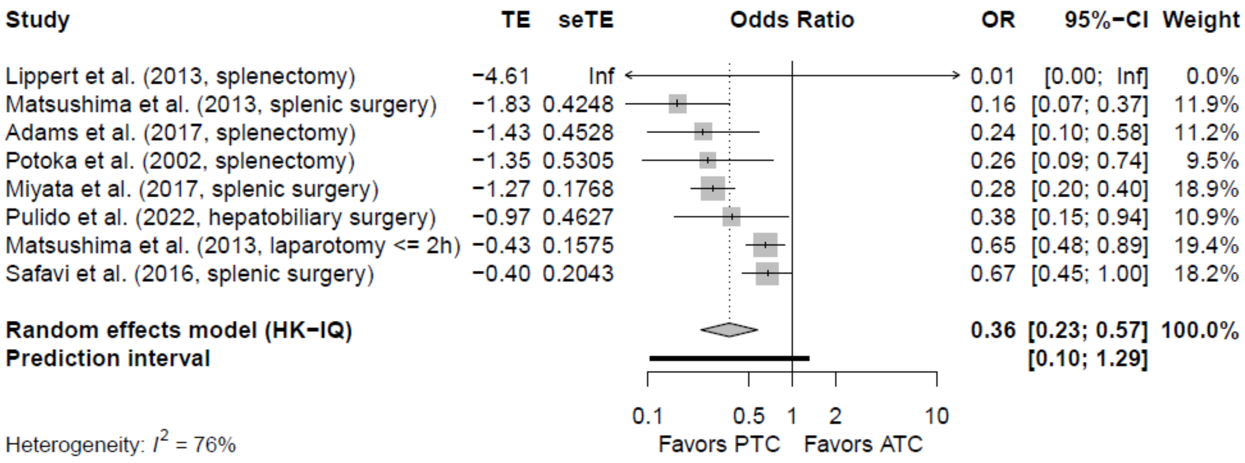

© 2023 Moore L et al. *JAMA Network Open.*

eFigure 27. Forest Plots Describing the Odds of Operative Management for Children Admitted to Hospitals Following Trauma Treated at Level I Pediatric Trauma Centers (PTCs) vs Level I Adult Trauma Centers (ATCs)

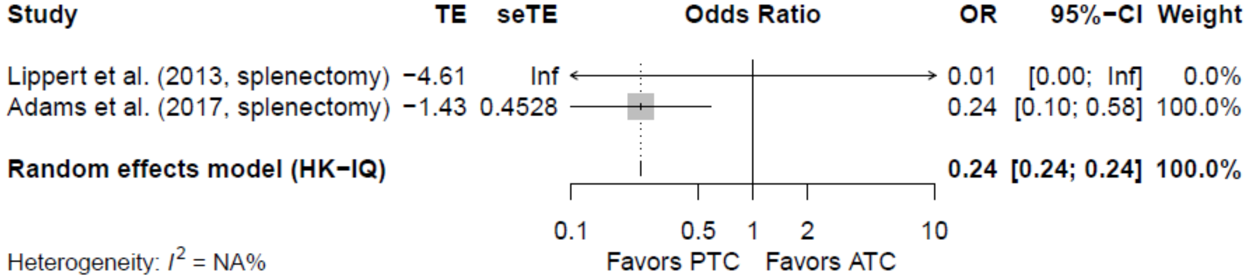

© 2023 Moore L et al. *JAMA Network Open.*

eFigure 28. Forest Plots Describing the Odds of Operative Management for Children Admitted to Hospitals Following Trauma Treated at Level I and II Pediatric Trauma Centers (PTCs) vs Level I and II Adult Trauma Centers (ATCs)

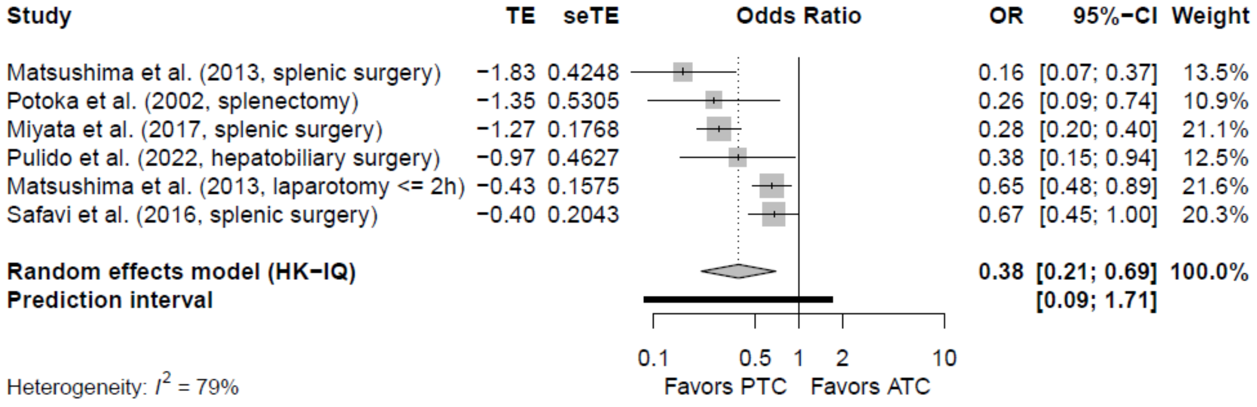

© 2023 Moore L et al. *JAMA Network Open.*

eFigure 29. Forest Plots Describing the Odds of Operative Management for Children Admitted to Hospitals Following Trauma Treated at Level I and II Pediatric Trauma Centers (PTCs) vs Level I and II Adult Trauma Centers (ATCs) With the Same Verification Organization

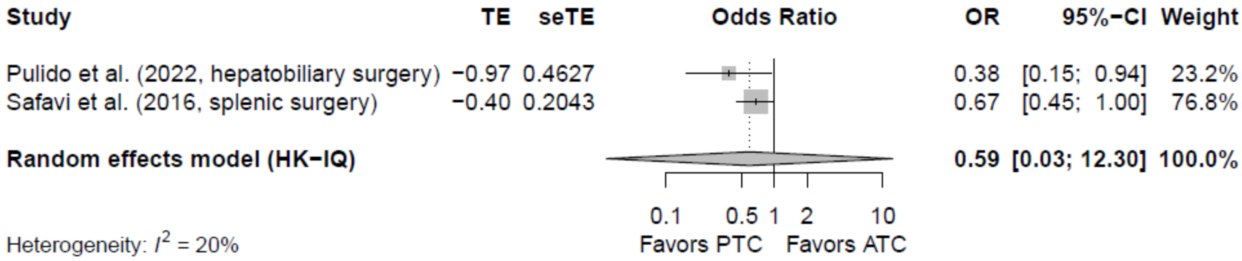

© 2023 Moore L et al. *JAMA Network Open.*

eFigure 30. Forest Plots Describing the Odds of *Mortality* for Children Admitted to Hospitals Following Trauma Treated at Pediatric Trauma Centers (PTCs) vs Combined Adult and Pediatric Centers (CTCs)

Study	TE	seTE	OR	95%−CI	Weight
Webman et al. (2016)	−1.90	0.5823	0.15	[0.05; 0.47]	3.4%
Evans et al. (2021)	−0.65	0.4193	0.52	[0.23; 1.18]	5.5%
Stephenson et al. (2022)	−0.60	0.2061	0.55	[0.37; 0.82]	11.3%
Hatchimonji et al. (2020)	−0.58	0.1315	0.56	[0.43; 0.72]	14.1%
Swendiman et al. (2021)	−0.54	0.3537	0.58	[0.29; 1.16]	6.8%
Sathya et al. (2015)	−0.37	0.1637	0.69	[0.50; 0.95]	12.9%
Miyata et al. (2017)	−0.30	0.2425	0.74	[0.46; 1.19]	10.0%
Osler et al. (2001)	0.02	0.1065	1.02	[0.83; 1.26]	15.0%
Sheff et al. (2022)	0.05	0.2936	1.05	[0.59; 1.87]	8.4%
Bardes et al. (2018)	0.20	0.2383	1.22	[0.76; 1.95]	10.1%
Lewit et al. (2022)	0.67	0.6824	1.96	[0.51; 7.47]	2.6%
**Random effects model (HK−IQ)**			**0.73**	**[0.53; 0.99]**	**100.0%**
**Prediction interval**				**[0.36; 1.48]**	

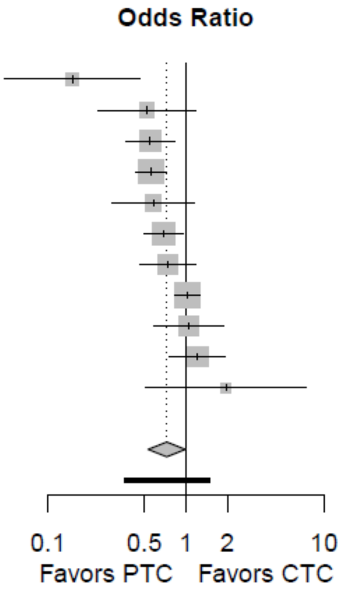

Heterogeneity: $I^2 = 69\%$

© 2023 Moore L et al. *JAMA Network Open.*

eTable 6. Results Not Included in Meta-Analyses[1]

First author, year	Intervention (I)	Comparator (C)	Outcomes	Results	
**Mortality/morbidity**				**OR (95% CI)**	
Swendiman, 2021b	PTC	ATC, CTC	Mortality	0.58 (0.29-1.16)	
Khalil, 2021	PTC	ATC	Discharge home	1.5 (1.1-1.7)	
Miyata, 2017	PTC	ATC	Non home discharge	0.83 (0.67-1.04)	
Webman, 2016	PTC	ATC, CTC (L2)	Discharge to rehabilitation	0.76 (0.38-1.56)	
**Resource use**				**Mean (I)**	**Mean (C)**
Walther, 2014	PTC L1 & L2	ATC L1	LOS (blunt)	4.4	4.6[2]
Walther, 2014	PTC L1 & L2	ATC L1	LOS (penetrating)	6.1	4.6[2]
Walther, 2014	PTC L1 & L2	ATC L1	ICU LOS (blunt)	0.5	0.7[2]
Walther, 2014	PTC L1 & L2	ATC L1	ICU LOS (penetrating)	0.5	0.7[2]
Sheff, 2022	PTC	ATC	ICU admission	OR: 0.47 (0.36-0.61)	
Sheff, 2022	PTC	CTC	ICU admission	OR: 0.52 (0.41-0.67)	
Khalil, 2021	PTC	ATC	ICU free days	RR: 1.22 (1.16-1.23)	
Khalil, 2021	PTC	ATC	Ventilator free days	RR: 1.07 (1.01-1.15)	
Walther, 2014	PTC L1 & L2	ATC L1	Ventilator days (blunt)	0.3	0.4[2]
Walther, 2014	PTC L1 & L2	ATC L1	Ventilator days (penetrating)	0.2	0.3[2]
Alexander, 2019	PTC	ATC	Average hospital charges<br>Average professional charges	$72,700<br>$14,636	$121,935[3]<br>$30,807[3]
**Processes of care**				**OR (95% CI)**	
Marin, 2015	PTC L1	Non-designated	Any CT<br>Head CT<br>C-spine CT<br>Chest CT<br>Abdominal CT	1.15 (1.09-1.21)<br>0.93 (0.88-0.98)<br>0.23 (0.19-0.27)<br>0.43 (0.29-0.64)<br>1.23 (1.01-1.50)	
Derderian, 2022	PTC	ATC	Blood product transfusion	5.8%	35.3%[3]
Swendiman, 2020	 PTC<br>PTC<br>PTC<br>PTC	 ATC<br>CTC<br>ATC<br>CTC	Interventional radiology:<br>Splenic injury<br>Splenic injury<br>Splenic, hepatic, renal injuries<br>Splenic, hepatic, renal injuries	 0.11 (0.05-0.28)<br>0.10 (0.04-0.27)<br>0.21 (0.12-0.37)<br>0.21 (0.11-0.38)	
Killien, 2022	PTC	ATC	Tracheostomy	0.55 (0.45-0.68)	
Killien, 2022	PTC	CTC	Tracheostomy	0.68 (0.55-0.83)	
Swendiman, 2021	PTC	ATC	Angioembolization, splenectomy	0.11 (0.14-0.19)	
Filipescu, 2020	PTC	ATC, CTC	Operative management	0.19 (0.11-0.32)	
Prieto, 2020	PTC	CTC	Surgical amputation	0.28 (0.1-1.4)	
Yung, 2020	PTC	ATC, CTC	Operative intervention	0.42 (0.36-0.48)	
Miyata, 2017	PTC	ATC, CTC	Total splenectomy	0.28 (0.20-0.40)	
Matsushima, 2013	PTC	ATC	Splenectomy	0.16 (0.07-0.37)	

PTC, pediatric trauma center; ATC, adult trauma center; CTC, combined trauma center; OR, odds ratio; CI, confidence intervals; LOS, length of stay; ICU, intensive care unit; RR, Rate Ratio; CT, computed tomography

[1]Could not be included because there were fewer than two studies with same exposure, comparator, and outcome

[2]$p>0.05$

[3]$p<0.05$

© 2023 Moore L et al. *JAMA Network Open.*
