# Supplementary material for: Pediatric vs Adult or Mixed Trauma Centers in Children Admitted to Hospitals Following Trauma: A Systematic Review and Meta-Analysis
Source: JAMA Netw Open. 2023 Sep 18;6(9):e2334266. doi: 10.1001/jamanetworkopen.2023.34266 (PMC10507486; doi:10.1001/jamanetworkopen.2023.34266)
Supplement: Supplement 2. — Data Sharing Statement [file jamanetwopen-e2334266-s002.pdf]

Data Sharing Statement

Moore. Pediatric vs Adult or Mixed Trauma Centers in Children Admitted to Hospitals Following Trauma. *JAMA Netw Open*. Published September 18, 2023.
doi:10.1001/jamanetworkopen.2023.34266

Data
Data available: Yes
Data types: Data (not involving human participants)
How to access data: Requests for data may be sent to lynne.moore@fmed.ulaval.ca
When available: With publication

Supporting Documents
Document types: Statistical/analytic code
How to access documents: Requests for analytic code may be sent to lynne.moore@fmed.ulaval.ca
When available: With publication

Additional Information
Who can access the data: The data will be made available to researchers/trainees whose proposed use of the data has been approved
Types of analyses: The data will be made available for any purpose
Mechanisms of data availability: The data will be made available without investigator support
